# Meta-inflammation in type 2 diabetes mellitus: unveiling the role of aberrant CD4^+^ T cells and pro-inflammatory cytokine networks

**DOI:** 10.3389/fimmu.2025.1603484

**Published:** 2025-09-15

**Authors:** Shubham K. Shaw, Soumya Sengupta, Rohila Jha, Chandrasekhar Pattanaik, Harapriya Behera, Prakash K. Barik, Dayanidhi Meher, Rajlaxmi Sarangi, Satish Devadas

**Affiliations:** ^1^ Department of Infectious Disease Biology, Biotechnology Research and Innovation Council (BRIC)-Institute of Life Sciences, Bhubaneswar, Odisha, India; ^2^ Department of Infectious Disease Biology, Regional Centre for Biotechnology (RCB), Faridabad/Gurgaon, Haryana, India; ^3^ Kalinga Institute of Medical Sciences, Bhubaneswar, Odisha, India

**Keywords:** T2DM, meta-inflammation, CD4+ T-cells, cytokines, TNF-α, STAT3, flow cytometry, antibody

## Abstract

This study aimed to investigate the causal or casual relation between dysregulated glucose metabolism and meta-inflammation in type 2 diabetes mellitus (T2DM), and more importantly the mediators and cellular sources for this meta-inflammation. We examined whether T2DM meta-inflammation is driven by aberrant, inflamed T-helper cells and if there was a direct link to HbA1c levels. Flow cytometry data revealed TNF-α−secreting effector CD4^+^ T cells as key contributors to inflammation, while memory T cells secreting GM-CSF and IL-17 escalated and maintained meta-inflammation. Crucially, these cytokines were present even in the “resting CD4^+^ T cells,” reflecting an aberrant, low-grade, chronically activated, and inflamed immune system. Significantly, higher antibody isotype levels further substantiated these findings as proof of concept for sustained and inflamed APC-T cell-B cell nexus. while reduced IL-10 levels reflected a shift towards pro-inflammatory bias. Functional assays, phospho-protein expression, *ex-vivo* inhibitor studies, and confocal microscopy confirmed that basal meta-inflammation in T2DM is exclusively mediated by multiple T-helper cell phenotypes via the TNF-α/STAT-3-signaling axis. Plasma cytokine and antibody isotyping were profiled using multiplex immunoassays from undiluted plasma. Taken together, these findings suggest that unchecked cytokine secretion, inflamed T-helper subsets, unwarranted antibody isotypes, and so forth, may contribute to organ damage by further amplifying innate and adaptive immune responses. Monitoring inflammatory cytokines, antibody isotypes, and T-helper cell subsets could significantly mitigate organ damage in T2DM, offering a more comprehensive approach to disease management. Thus, this study highlights the importance of not only achieving metabolic control during T2DM treatment but also monitoring and regulating immune homeostasis.

## Introduction

Type 2 diabetes mellitus (T2DM) has been increasingly recognized as a multi-organ, metabolic, and inflammatory disease characterized by persistent hyperglycemia, chronic low-grade inflammation, and a dysfunctional immune system (1). Meta-inflammation in T2DM is well documented and associated with microvascular and macrovascular complications such as chronic kidney disease, retinopathy, atherosclerosis, cardiomyopathy, hepatopathy, neuropathy, diabetic foot ulcerations, and so forth (2–5). Despite this generalized understanding of subclinical inflammation involved in multi-organ damage in T2DM, the exact and/or specific cellular sources and mediators driving meta-inflammation in T2DM remain poorly defined. Additionally, the lack of correlation between glycated hemoglobin (HbA1c) levels, and the negative impact of an aberrant inflammatory immune response diminishing effective therapeutic interventions with major organopathies implicated is very poorly understood (6–8). Most importantly, the presumption of good health with acceptable clinical and biochemical laboratory parameters, with the exception of higher HbA1c, has serious immunological consequences when underlying meta-inflammation is not addressed (9). Chronic T2DM has immune implications bordering on mild to severe depending on dysregulated glucose, Hb1Ac levels, and so forth, and is reflected more severely during an infection or injury (10–12).

While T2DM meta-inflammation has both innate and adaptive immune components (13, 14), we have previously shown that T2DM patients with COVID-19 had elevated cytokine levels, higher non-protective antibody levels, and an altered immune cell profile, correlating with meta-inflammation, with a definite higher adaptive immune bias (14). CD4^+^ T-helper cells with elevated pro-inflammatory markers have been shown to infiltrate both visceral and subcutaneous adipose tissue (15). Additionally, studies using obese mouse models have illustrated the involvement of T cells in adipose tissue inflammation (16–18), suggesting the need for further investigation into T-cell roles in T2DM patients.

Research on immune cell involvement in T2DM has largely focused on innate immune cells, such as macrophages, dendritic cells, and neutrophils (19–21). However, their short lifespan and lack of memory function limit their role in sustaining long-term low-grade inflammation (22). In contrast, adaptive immune cells, particularly T cells, may provide a comprehensive view of inflammatory processes underlying T2DM (23, 24). Chronic meta-inflammation in T2DM has been linked to certain immune mediators, such as TNF-α, IL-6, and IL-1β and altered signaling pathways including NF-κb and NLRP3 inflammasome (25–27). However, other cytokines, including GM-CSF, IL-17, and IFN-γ and signaling pathways, such as STATs and MAPKs, have not been extensively studied, though they may offer valuable insights into disease progression.

The study recruited 100 T2DM patients and 40 healthy controls from KIMS hospital, Bhubaneswar, along with clinical data such as HbA1c, glucose levels, CRP, and lipid profile following ethical approval and informed consent. Neat plasma samples were analyzed for cytokines, chemokines, soluble receptors, growth factors, and antibody isotypes using multiplex assays. Peripheral blood mononuclear cells (PBMCs) were isolated, stimulated, and assessed for intracellular proteins along with phosphorylated STATs by flow cytometry.

## Materials and methods

### Clinical details and demographics of T2DM patients

Ethical clearance was obtained prior to sample collection and the authors concerned declare no conflict of interest. One hundred DM confirmed subjects (HbA1c > 6) were recruited from KIMS Hospital, Bhubaneswar, Odisha from June 2021 to October 2023 along with 40 age and sex-matched healthy subjects. Laboratory parameters such as HbA1c, fasting plasma glucose (FPG), Post Prandial Plasma Glucose (PPPG), estimated average glucose, C-reactive protein (CRP), Lipid profile, and so forth were analyzed and collected from the Department of Biochemistry, Central Laboratory (NABL accredited), KIMS Hospital. All the T2DM participants recruited were on anti-diabetic medication and were free of co-morbidities or other known disease or infections. Clinical details, drug history were collected from individuals. Signed Informed Consent forms were obtained from all participants in the study. 11% of T2DM patients were on insulin, while 89% were on oral hypoglycemics (**Table 1**) such as Metformin, Glimepiride, Gliptins, Vidagliptin, Sitagliptin, Linagliptin, Voglibose, Dapagliflozin, Empagliflozin.

**Table 1:** Baseline demographics and clinical characteristics of healthy control subjects and T2DM patients.

Characteristics	HC (40)	T2DM (100)
Age	35 ± 8	53.13 ± 10.46
Female	45%	38%
Male	55%	62%
HbA1c (< 5.7)	< 5.5	7.6 ± 1.5
Random plasma Glucose (mg/dl) (70–140)	N.R	128 ± 26
FPG (mg/dl) (70–100)	N.R	147 ± 71
PPPG (mg/dl) (70–140)	N.R	213 ± 77
Avg. blood Glucose	N.R	175 ± 44
Total cholesterol (mg/dl) (< 200)	N.R	180 ± 45
HDL (mg/dl) (> 40)	N.R	46 ± 20
LDL (mg/dl) (< 130)	N.R	97 ± 34
HDL ratio	N.R	4.4 ± 1.9
Triglycerides (mg/dl) (< 150)	N.R	192 ± 100
Serum Creatine (mg/dl) (0.5–1.3)	N.R	1.22 ± 1.21
Serum Sodium (mg/dl) (136.0–146.0)	N.R	137.05 ± 4.45
Serum Globulin	N.R	3.1 ± 0.6
Serum Albumin (mg/dl) (3.5–5.0)	N.R	4.11 ± 0.38
SGPT (U/L) (5.0–40.0)	N.R	31 ± 23
SGOT (U/L) (0.0–40.0)	N.R	33 ± 32
Serum Alkaline Phosphatase (U/L) (35–129)	N.R	95 ± 33

Medications:

Insulin: 11%.

Oral Hypoglycemics: 89%.

(Metformin, Glimepiride, Gliptins; Vilda, Sita, Lina, Voglibose, Dapagliflozin, Empagliflozin).

HC, Healthy controls; T2DM, Type 2 Diabetes mellitus; Hb1AC, glycated hemoglobin; FPG, Fasting Plasma Glucose; PPPG, Postprandial Plasma Glucose; HDL, High-density lipoprotein; LDL, low-density lipoprotein; CRP,C-reactive protein; SGOT, serum glutamic-oxaloacetic transaminase; SGPT, serum glutamic-pyruvic transaminase; N.R, Normal range. Age values are represented as %. Other clinical parameters are represented as Mean ± SD.

### Plasma cytokines, chemokines, soluble receptors, growth factors, and antibody isotypes detection assay

Neat plasma derived from T2DM patients (*n*=30) and healthy controls (*n*=20) were run in duplicates to measure 46 different analytes including cytokines, chemokines, soluble receptors, and growth factors using a Human ProcartaPlex Mix & Match 46-Plex kit (Cat. No PPX-46-MX324DE, Invitrogen, Vienna, Austria), based on manufacturer’s instructions. Another set of neat plasma from same 30 T2DM patients and 20 healthy volunteers, were analyzed by ProcartaPlex Human Antibody Isotyping Panels (Cat. No EPX070-10818-901, Invitrogen, Vienna, Austria). The samples were acquired in a Bio-Plex 200 system, and analyte concentrations were calculated using Bio-Plex manager software with a five-parameter (5PL) curve-fitting algorithm applied for standard curve calculation (28, 29).

### PBMC isolation, surface and intracellular staining for flow cytometry

PBMCs were isolated via density gradient centrifugation from T2DM patients (20) and healthy volunteers (*n*=20). Isolated cells were then activated using PMA (20 ng/ml) and Ionomycin (1 μg/ml) for 8h. Brefeldin (5 μg/ml) and Monensin (2 μmol) were added during the last 4h and dead cells were excluded using a Zombie Fixable NIR or Aqua Dye Kit (Biolegend, San Diego, CA, USA). Intracellular cytokine staining was performed with fluorochrome tagged surface markers, cytokines, and transcription factors using a Cytofix/Cytoperm Fixation/Permeabilization Solution Kit (BD Biosciences, San Jose, CA, USA). In contrast, transcription factor staining was performed using a FOXP3 staining buffer set (eBioscience, San Diego, CA, USA) according to the manufacturer’s instructions followed by target specific fluorochrome tagged antibody staining for 30 min and acquired in BD LSR Fortessa (30, 31). The gating strategy is detailed in the supplementary figures (**Supplementary Figure S1**).

### Intracellular phospho-protein staining

For pSTAT expression, CD4^+^ T cells from T2DM patients (*n*=10) and healthy volunteers (*n*=10) were stimulated with PMA (20 ng/ml) and Ionomycin (1 μg/ml) for 6h, fixed with 2% formaldehyde for 30 min at RT, permeabilized with 1% Triton-X and 90% Methanol, washed with eBiosciences Perm wash buffer. Cells were then stained with fluorochrome-labelled phospho STAT antibodies for 30 min, washed, and acquired in BD LSR Fortessa (32–34). The gating strategy used for analysis is provided in **Supplementary Figure S2**.

### *Ex-vivo* inhibitor studies

For inhibitor studies, 30 samples of T2DM PBMCs were stimulated with the mentioned different inhibitors along with PMA (20 ng/ml) and Ionomycin (1 μg/ml) and 4h post-stimulation, Brefeldin (5 μg/ml) and Monensin (2 μmol), and incubated for another 12h. The details of the inhibitors and their concentration used are provided in the **Supplementary Tables S1** and **S2** and the details of flurochrome tagged antibodies are provided in **Supplementary Table 3**. After 16h, cells were washed, and dead cells were excluded using a Zombie Fixable Aqua Dye Kit (BioLegend, San Diego, CA, USA). Intracellular staining was performed as described previously, and data was acquired on a BD, LSR Fortessa (30, 31, 34, 35). The gating strategy used for analysis is provided in **Supplementary Figure S3**.

### Confocal microscopy

For pSTATs nuclear localization studies, CD4^+^ T cells from PBMCs of 10 T2DM patients were stimulated with PMA/Ionomycin for 6h and stained for pSTAT1 and pSTAT3. The cells were then smeared on pre-coated Poly-L-Lysine slides with anti-fade and DAPI. TCS SP5 Leica confocal microscope was used to visualize pSTAT staining using 488 nm and 640 nm lasers (35).

### Th1, Th2, and Th17 cells differentiation from healthy controls

CD4^+^ T-cell population was derived from PBMCs using Dynabeads Human CD4 T-cell kit. Cell purity was ascertained to be ~90% (**Supplementary Figure S4**) and the isolated cells were cultured in RPMI 1640, supplemented with 10% Foetal Bovine serum, Australian origin, 100 U/ml Penicillin, 100 mg/ml Streptomycin and 50 mM 2β-Mercaptoethanol. For Th1 differentiation, cells were plated on pre-coated αCD3 (1 μg/ml) and αCD28 (2 μg/ml) along with neutralizing antibody αIL-4 (10 μg/ml) and cytokines IL-12 (10 ng/ml) and IL-2 (100 IU/ml). For Th2 differentiation, cells were plated on pre-coated αCD3 (1 μg/ml) and αCD28 (2 μg/ml) along with the neutralizing antibodies αIL-12 (10 μg/ml), αIFN-γ (10 μg/ml) and cytokines IL-4 (20 ng/ml) and IL-2 (100 IU/ml). For Th17 differentiation, cells were plated on pre-coated αCD3 (1 μg/ml) plates at 1 million per ml density, αCD28 was added in the soluble form (2 μg/ml) along with neutralizing antibodies, αIFN-γ (10 μg/ml), αIL-4 (10 μg/ml), and cytokines IL-6 (25 ng/ml), IL-21 (25 ng/ml), IL-23 (25 ng/ml), IL-1β (15 ng/ml), TGF-β (5 ng/ml). Th1 and Th2 cultures were activated for 5 days followed by resting for 2 days while Th17 cultures were activated for 7 days (35, 36). After 7 days all the cells were washed with RPMI 1640, and used for subsequent experiments. The gating strategy used for analysis is provided in **Supplementary Figure S5**.

### Design of statistical analysis

Non-parametric tests (such as Mann-Whitney tests, Kruskal Wallis tests, and Two-way ANOVA) and parametric test (paired t test) were performed using GraphPad Prism 8.0.1.

## Results

### Elevated inflammatory and antibody profile in T2DM patients

**Figure 1:**
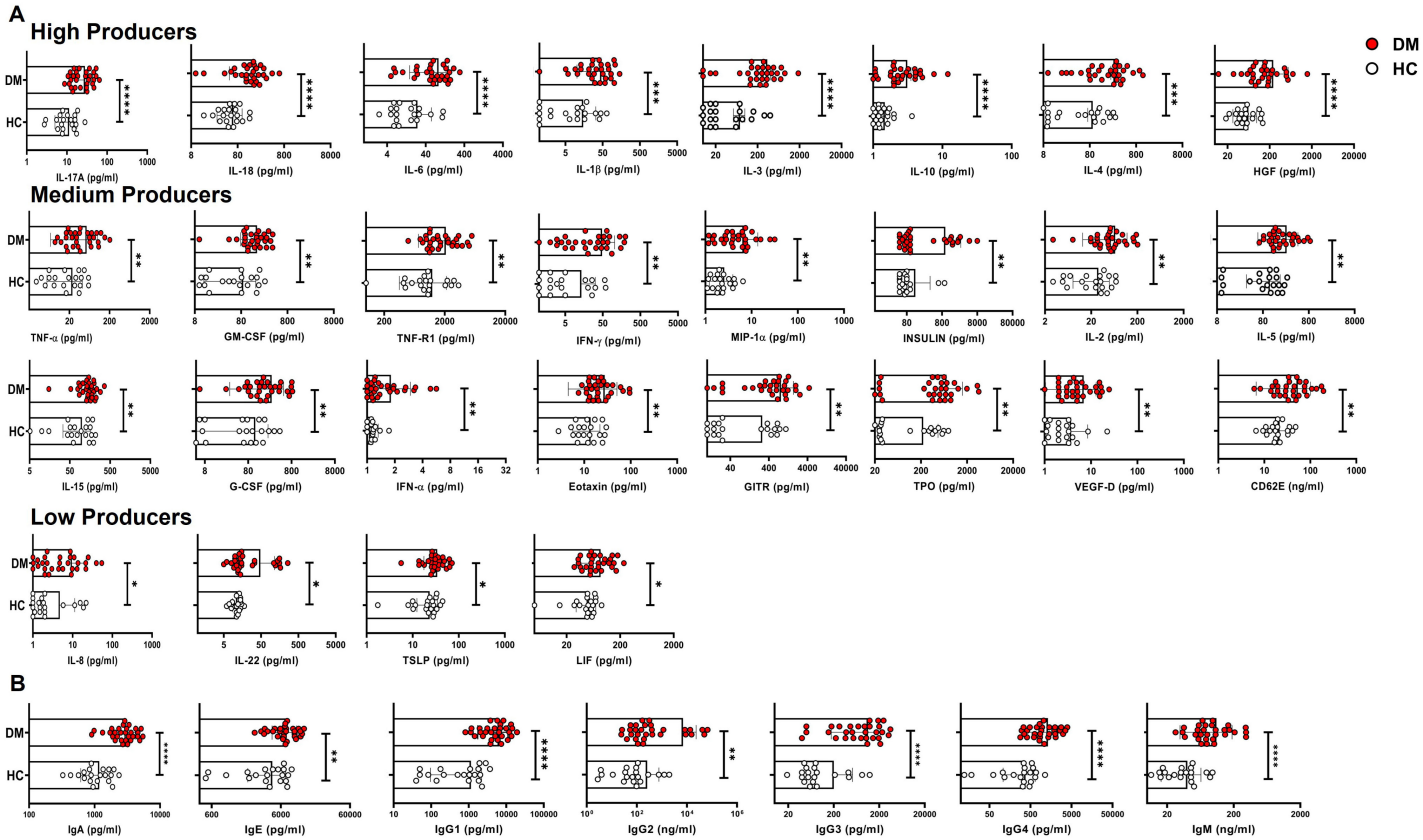
Plasma cytokine and antibody levels and biochemical and immune correlate in T2DM patients. Differential augmented expression levels (high, medium and low producers) of the cytokines, chemokines, growth factors and soluble receptors in plasma isolated from peripheral blood of T2DM patients (*n*=30) vs. healthy controls (*n*=20). IL-17A, IL-18, IL-6, IL-1β, IL-3, IL-4, IL-10, and HGF were highly elevated in T2DM patients compared to healthy controls and designated as high producers. TNFα, TNF-R1, GM-CSF, IFN-γ, MIP-1α, IL-5, IL-2, Insulin, IL-15, G-CSF, IFN-α, TPO, VEGF-D, Eotaxin, GITR, and CD62E were moderately elevated in T2DM patients when compared to healthy controls and designated as medium producers. IL-6, IL-22, LIF, and TSLP were slightly elevated in T2DM patients when compared to healthy controls and designated as low producers. **(A)**. Increased expression of the circulating antibodies such as IgG1, IgA, IgG2, IgG3, IgM, IgG4, and IgE in plasma isolated from peripheral blood of T2DM patients (*n*=30) vs. healthy controls (*n*=20) **(B)**. Error bar in the above bar diagrams indicates SD. Mann–Whitney U Test was performed to compare between the two groups; *p* < 0.05 was considered statistically significant (*); *p* < 0.01 was considered to be very significant (**); *p* < 0.001 was considered to be highly significant (***); *p* < 0.0001 was considered extremely significant (****).

To assess the extent of systemic inflammation, we examined for various pro-inflammatory markers in the plasma of T2DM patients. This included assessing cytokines, chemokines, and other inflammatory mediators known to contribute to metabolic dysregulation and immune activation in diabetes. Amongst 46 different analytes, T2DM patients displayed significantly higher inflammatory proteins, including antibody isotypes, than healthy controls. We observed elevated levels of 27 plasma proteins, including 18 cytokines, three chemokines, four growth factors and two soluble receptors, and labelled them as high, medium, and low expressers (**Figure 1A**) based on comparison with healthy controls. Among the high producers we observed statistically significant levels of IL-17, IL-18, IL-6, IL-3, IL-10, HGF, IL-1β, and IL-4. Among the medium producers, we observed significant levels of TNF-α, VEGF-D, GMCSF, MIP-1α, GITR, GCSF, CD62E, TNFRI, TPO, insulin, IL-5, IFN-α, IL-2, and eotaxin, IL-15, and IFN-γ. In the low producers, we observed significant levels of IL-8, TSLP, IL-22, and LIF. We further examined seven different antibody isotypes in T2DM patients to validate if increased cytokine levels for a sustained period of time between 45 and 60 days could lead to an increase in circulating antibody isotypes, as suggested by several reports (14, 37). Not surprisingly, all seven antibody isotypes analyzed, displayed a significant statistical increase in T2DM patients when compared with healthy controls, including IgG1, IgG3, IgG4, IgA, IgM, IgE, and IgG2 (**Figure 1B**).

### Resting T cells as a cellular source of pro-inflammatory cytokines

**Figure 2:**
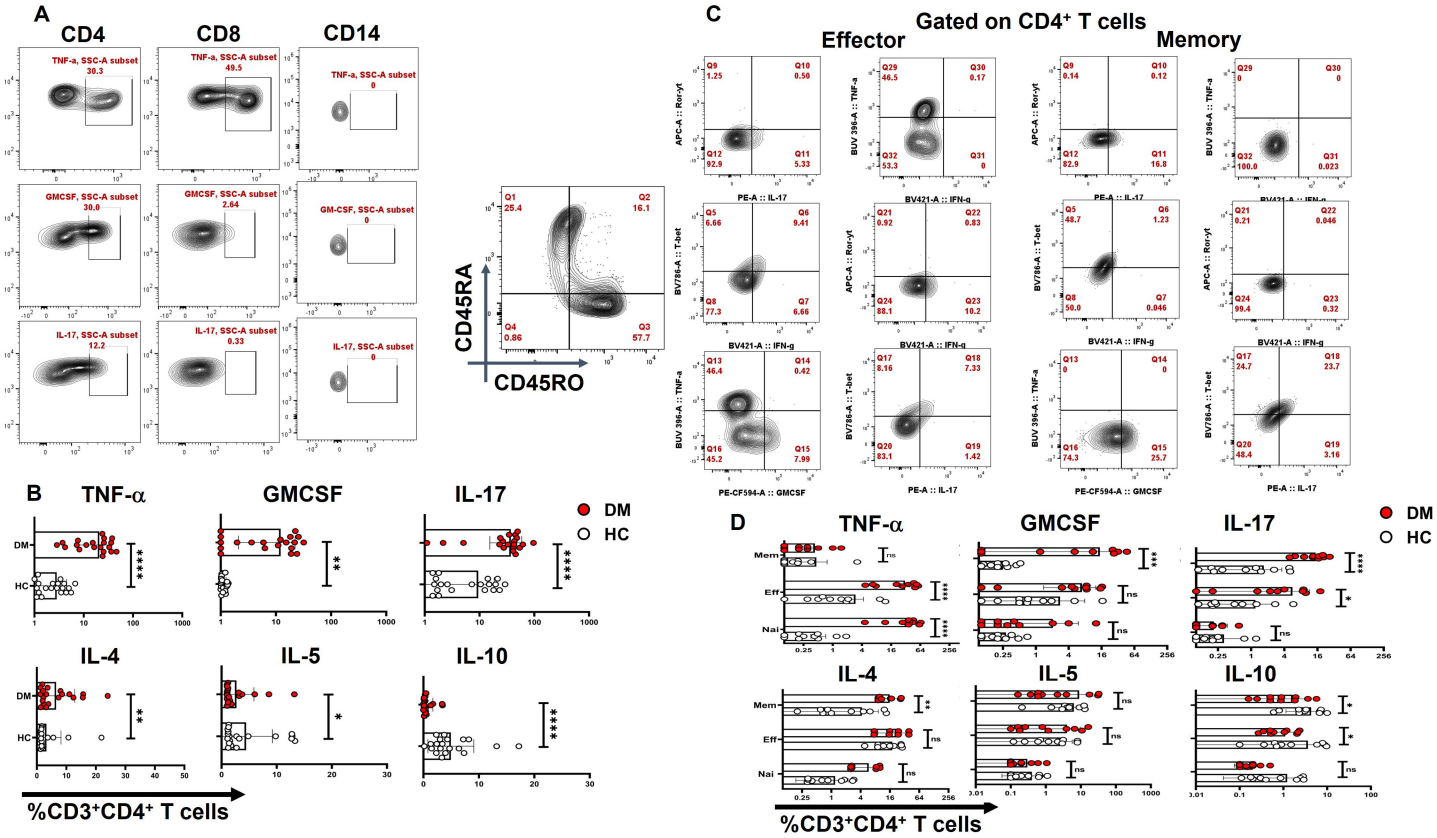
CD4^+^ T-cell cytokine secretion in resting T cells. “Resting” state CD4^+^ cells of T2DM patients (n=20) exhibit significantly higher basal levels of pro-inflammatory cytokines, such as IL-17, TNF-α, and GM-CSF followed by higher secretion from CD8^+^ and CD14^+^ cells as opposed to healthy controls as represented in flow cytometry plots **(A)** and graphical plots **(B)**. Amongst pro-inflammatory cytokines, effector and naive resting CD4^+^ T cells secrete elevated levels of TNF-α and memory compartment predominantly secrete GM-CSF and IL-17. Anti-inflammatory cytokines such as IL-4 expression is significantly higher and from the memory compartment, IL-10 expression is lower in both effector and memory compartments compared to HCs **(C, D)**. The error bar in the above bar diagrams indicates SD. Mann–Whitney U Test was performed to compare the two groups **(B)**, Two-way ANOVA was performed to compare the different T-cell compartments between DM and HC **(D)**, *p* < 0.05 was considered statistically significant (*); *p* < 0.01 was considered to be very significant (**); *p* < 0.001 was considered to be highly significant (***); *p* < 0.0001 was considered extremely significant (****).

In our next step, the cellular source of inflammatory cytokines in T2DM patients was analyzed with immune cell surface markers and the inflammatory cytokines to establish metabolic dysregulation, inflammatory cytokine bias, and aberrant immune cells. Amongst 20 T2DM patient samples analyzed, all displayed statistically significant higher basal levels of pro-inflammatory cytokines, such as IL-17, TNF-α, and GM-CSF, secreted by CD4^+^ T cells even in their “resting” state (**Figures 2A, B**), along with IL-4, IL-5, and IL-10, apart from the slightly higher secretion from CD8^+^ and CD14^+^ cells, and were completely absent in healthy controls. Further investigation revealed that TNF-α was secreted majorly from the effector compartment, and GM-CSF and IL-17 were found to be predominantly secreted from the memory compartment from resting CD4^+^ T cells (**Figures 2C, D**). These results strongly suggested that the altered T2DM metabolic status may have a deleterious impact on the immune system, potentially contributing to chronic inflammation and associated tissue and organ damage.

### Cytokine bias in T-helper subsets and its compartments

**Figure 3:**
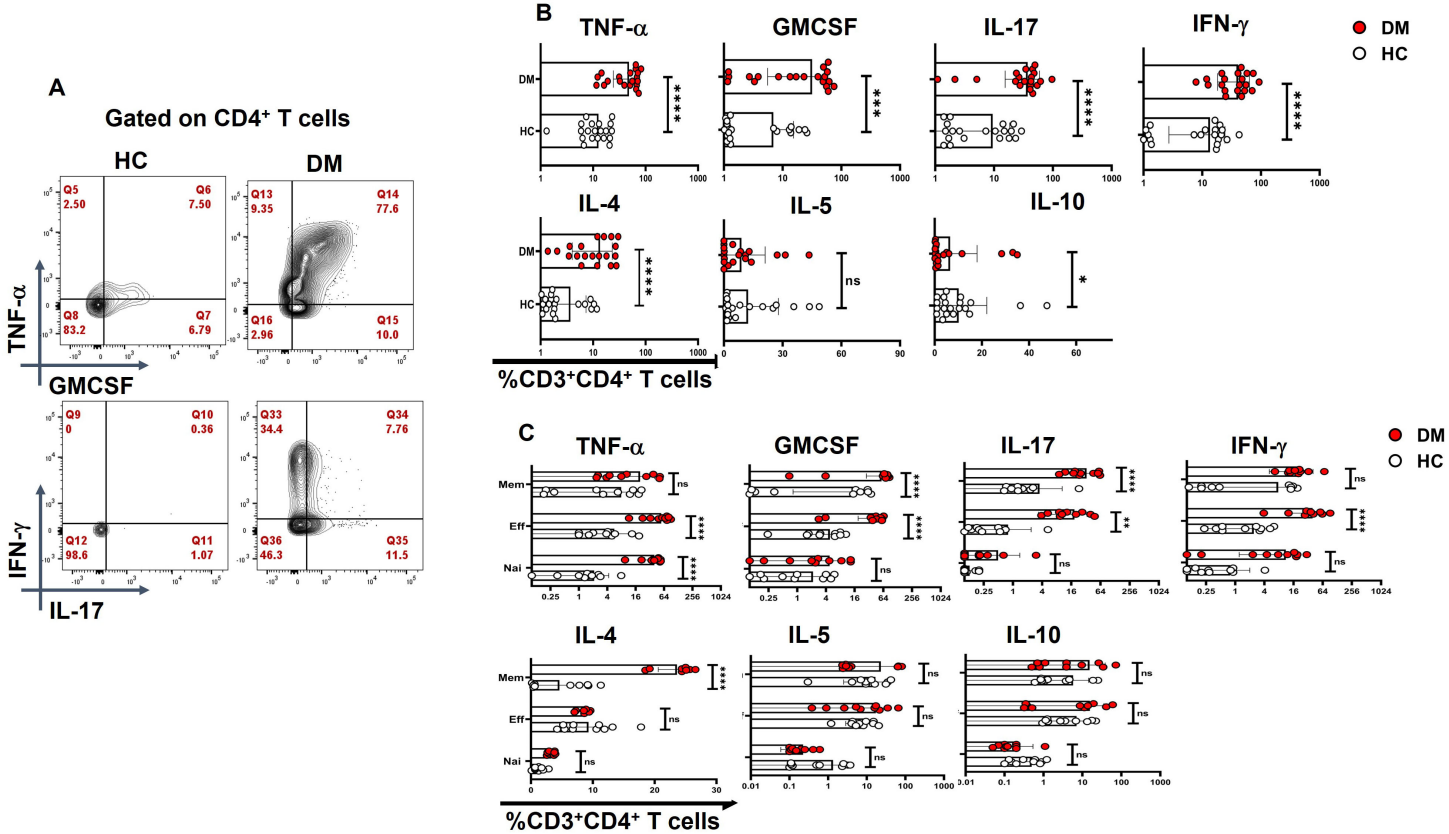
CD4^+^ T-cell cytokine secretion in activated T cells. “Activated” CD4^+^ T cells of T2DM show higher expression of inflammatory cytokines and anti-inflammatory cytokines such as IL-4 as compared to healthy controls represented by flow cytometry plots **(A)** and graphical plots **(B)**. Effector and memory compartments of “Activated” CD4^+^ T cells of T2DM shows higher expression of pro-inflammatory cytokines with naïve compartment also showing higher levels of TNF-α, however anti-inflammatory cytokines such as IL-4 expression is significantly from the memory compartment as compared to healthy controls represented by graphical plots **(C)**. The error bar in the above bar diagrams indicates SD. Mann–Whitney U Test was performed to compare the two groups **(B)**, Two-way ANOVA was performed to compare the different T-cell compartments between DM and HC **(C)**, *p* < 0.05 was considered statistically significant (*); *p* < 0.01 was considered to be very significant (**); *p* < 0.001 was considered to be highly significant (***); *p* < 0.0001 was considered extremely significant (****). ns, not significant.

Activation with PMA/Ion revealed an augmented level of pro-inflammatory cytokine release from CD4^+^ T cells of T2DM patients when compared with healthy controls, including TNF-α, GM-CSF, IFN-γ, and IL-17 (**Figures 3A, B**). Further analysis into specific T-helper cell compartments revealed that GMCSF and IL-17 were consistently higher in both effector and memory compartments, with TNF-α and IFN-γ being significantly higher in the effector compartment (**Figure 3C**). Interestingly, even the naïve compartment secreted TNF-α, establishing activated naïve T cells. Effector and memory T cells were gated with the help of surface markers CD45RA and CD45RO (**Supplementary Figure S6**). We also examined anti-inflammatory cytokine expression, including IL-4, IL-5, and IL-10 (**Figure 3B**). Notably, CD4^+^ T cells in T2DM patients secreted significantly higher levels of IL-4 (**Figure 3B**), predominantly from the memory compartment (**Figure 3C**), and no change was observed with IL-5, but IL-10 expression was found to be markedly lower in T2DM patients when compared to HCs (**Figure 3B**). At the same time, IL-5 and IL-10 levels did not exhibit any statistical significance between T2DM patients and HCs when compared within the T-cell compartments (**Figure 3C**). The absence of significance does not rule out its secretion, as evinced by isotype antibody secretions. We report an aberrant CD4^+^ T-cell phenotype in T2DM, characterized by the secretion of multiple pro-inflammatory cytokines, with a subset of double-positive TNF-α and GM-CSF cells that also secreted IFN-γ and IL-17 (**Supplementary Figures 7A, B**). This aberrant T-cell phenotype was predominantly observed in both effector and memory compartments (**Figure 3C**).

### TNF-α neutralization modulates inflammatory cytokine secretion

**Figure 4:**
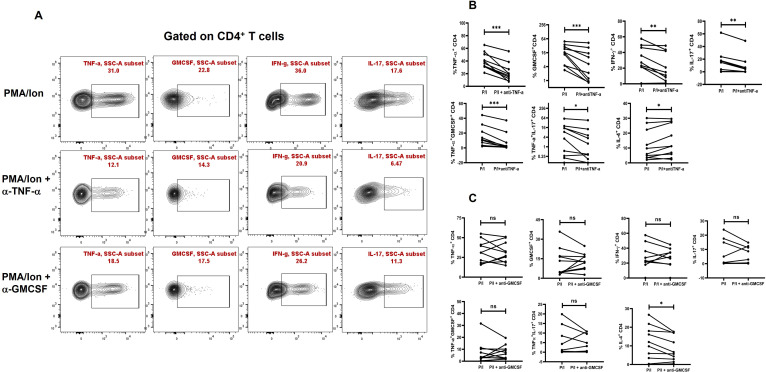
Modulation of cytokines with anti-TNF-α and anti-GMCSF treatment. Representative figures showing modulation of pro-inflammatory and anti-inflammatory cytokine expression in T2DM CD4^+^ T cells (*n*=20) derived from PBMCs with anti-TNF-a and anti-GMCSF treatment along with PMA/Ionomycin stimulation. PBMC derived CD4^+^ T cells displayed significant downregulation of Pro-inflammatory cytokines including TNF-α, GMCSF, IL-17 and IFN-γ with anti-TNF-α treatment as shown in the flowcytometric plots **(A)** and in graphical plots **(B)**. In addition, anti-GMCSF treatment did not show any significant differences in the pro-inflammatory cytokine expression as shown **(A, C)**. IL-4 expression increased significantly in PBMC derived CD4^+^ T cells with anti-TNF-α and decreased with anti-GMCSF treatment. The error bar in the above bar diagrams indicates SD. Paired t-Test was performed to compare the two groups **(B, C)**; *p* < 0.05 was considered statistically significant (*); *p* < 0.01 was considered to be very significant (**); *p* < 0.001 was considered to be highly significant (***). ns, not significant.

To assess the inflammatory potential of TNF-α on other pro-inflammatory cytokines, we conducted a neutralization assay using a TNF-α monoclonal antibody during activation of CD4^+^ T cells from T2DM patients. Following TNF-α inhibition, we observed a significant decrease in pro-inflammatory cytokines, including IL-17, IFN-γ, and GM-CSF. Additionally, the aberrant TNF-α^+^GM-CSF^+^ and TNF- α^+^IL-17^+^ secretion was also reduced significantly (**Figures 4A, B**) Among anti-inflammatory cytokines, there was a significant increase in IL-4^+^ cells (**Figure 4B**), while IL-5 and IL-10 levels remained unchanged upon TNF-α inhibition (data not shown). Apart from TNF-α inhibition, we also performed neutralization with GMCSF monoclonal antibody, which did not display any changes in the pro-inflammatory cytokine secretion in the T2DM patients, but a significant decrease in IL-4 was observed (**Figure 4C**).

### Correlation between biochemical and immune parameters

**Figure 5:**
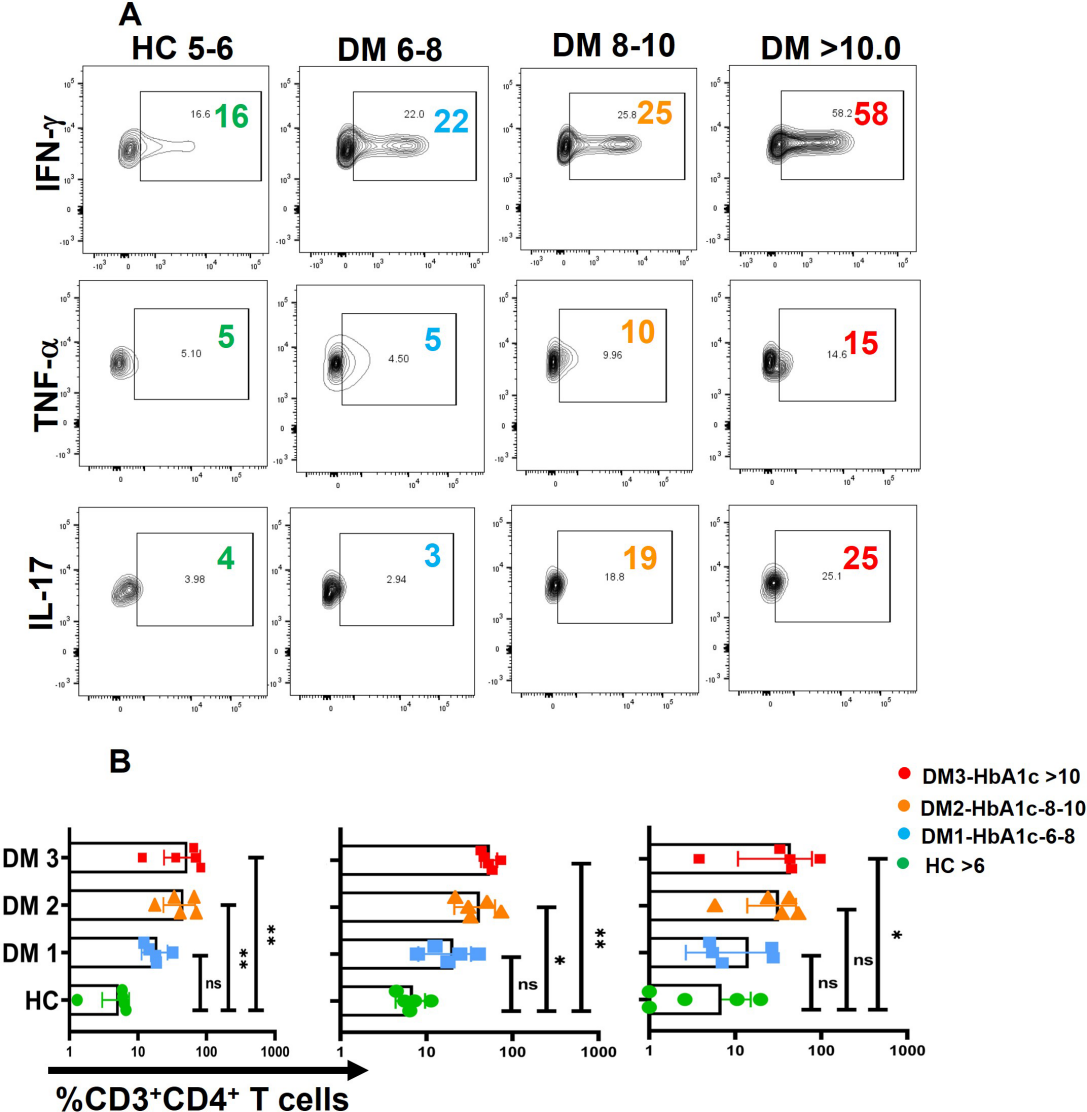
Correlation between biochemical parameters and Immune profile. A positive correlation was observed between HbA1c levels and pro-inflammatory cytokines namely TNF-α, IFN-γ and IL-17 in three different groups of T2DM patients with increasing levels of HbA1c labelled as DM1 (6-8), DM2 (8-10), and DM3 (<10) **(A)**, Error bar in the above bar diagrams indicates SD. Kruskal-Walis test was performed to compare the four groups **(B)**; *p* < 0.05 was considered statistically significant (*); *p* < 0.01 was considered to be very significant (**). ns, not significant.

While a majority of the proteins analyzed were significantly higher than healthy controls, the analyses clearly implied that a positive or negative correlation between the above proteins and HbA1c had to be considered with the caveat that HbA1c is a 90- to 120-day glucose dysregulation marker, while secreted cytokine would probably last between 1 and 3 days maximally in circulation. Nevertheless, despite the presence of circulating cytokines as shown in our data, along with our previous publication (14), these cytokines and chemokines are taken up or sensed by immune cells can trigger downstream effects, including modulation of antibody isotypes profiles and other immune responses. Therefore, we investigated the correlations between biochemical parameters and immune profiles to explore potential causal or associative relationships with chronic low-grade meta-inflammation in T2DM. To assess the impact of metabolic dysregulation on immune responses, we analyzed cytokine release from activated T cells across different patient groups and compared them with various biochemical markers. Our findings indicate that a substantial subset of the T2DM population exhibited a dysregulated carbohydrate and lipid profile, characterized by elevated HbA1c levels and increased activation of pro-inflammatory cytokines, including IFN-γ, TNF-α, and IL-17. These immune alterations were observed across three T2DM subgroups with progressively increasing HbA1c levels: DM1 (6–8), DM2 (8–10), and DM3 (>10) (**Figures 5A, B**). We did not find any correlation between any other anti-inflammatory cytokines and biochemical parameters. However, immune responses varied among patients, suggesting inter-individual heterogeneity, which may be influenced by factors such as disease progression, metabolic status, or medication use.

### Phospho-STAT expression and co-localization in DM CD4^+^ T cells

**Figure 6:**
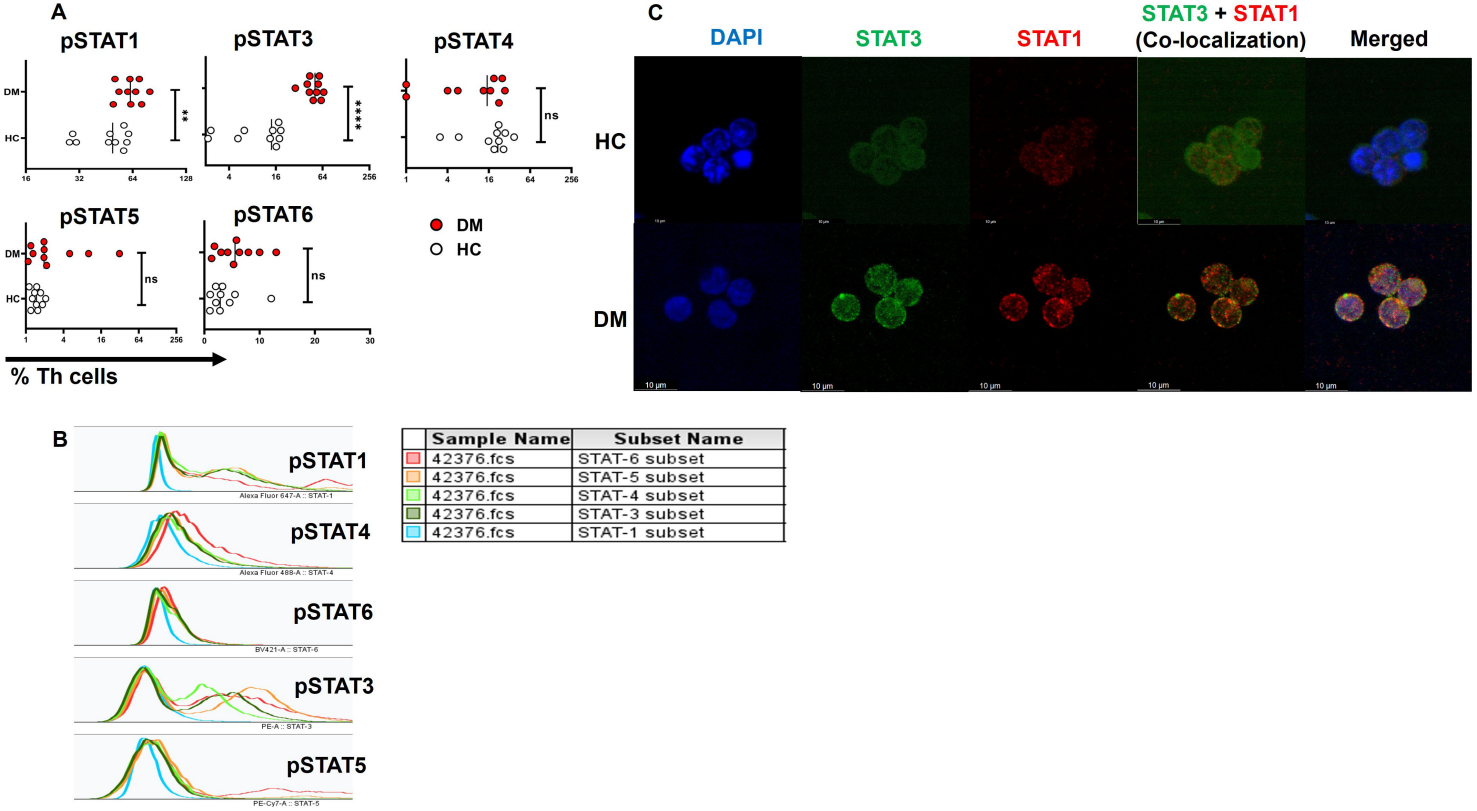
STAT protein expression and co-localization in CD4^+^ T cells of T2DM patients. Increased levels of pSTAT1 and pSTAT3 are observed in T-helper cells of T2DM patients (*n*=10) versus healthy controls **(A)**. Expression of pSTATs subsets (STAT1, 3, 4, 5, and 6) in CD4^+^ T cells of T2DM patients following activation with PMA/Ionomycin, represented via multiple overlay histogram plots **(B)**. Confocal images depict the co-localization of pSTAT1 and pSTAT3 in CD4^+^ T cells isolated from T2DM patients and compared to healthy controls **(C)**. The error bar in the above bar diagrams indicates SD. Mann–Whitney U Test was performed to compare the two groups **(A)**, *p* < 0.01 was considered to be very significant (**); *p* < 0.0001 was considered extremely significant (****). ns, not significant.

Finally, our objective was to investigate the signaling pathway and proteins potentially responsible for the elevated TNF-α expression, given that TNF-α was the primary pro-inflammatory cytokine expressed in T2DM patients, predominantly through the effector compartment. To address this, we examined the phosphorylation status of STAT proteins, including pSTAT1, pSTAT3, pSTAT4, pSTAT5, and pSTAT6, following activation with PMA/Ionomycin gated on CD4^+^ T cells (**Figure 6A**). Our findings revealed significantly higher levels of phosphorylated pSTAT1 and pSTAT3 in CD4^+^ T cells from T2DM patients compared to healthy controls (**Figures 6A, B**). Confocal microscopy further confirmed this result, showing nuclear co-localization of pSTAT1 and pSTAT3 in CD4^+^ T cells from T2DM patients but not in healthy controls (**Figure 6C**). Phosphorylation of other STAT proteins did not show significant differences between the two groups.

### Role of STAT3 in TNF-α-mediated inflammation

**Figure 7:**
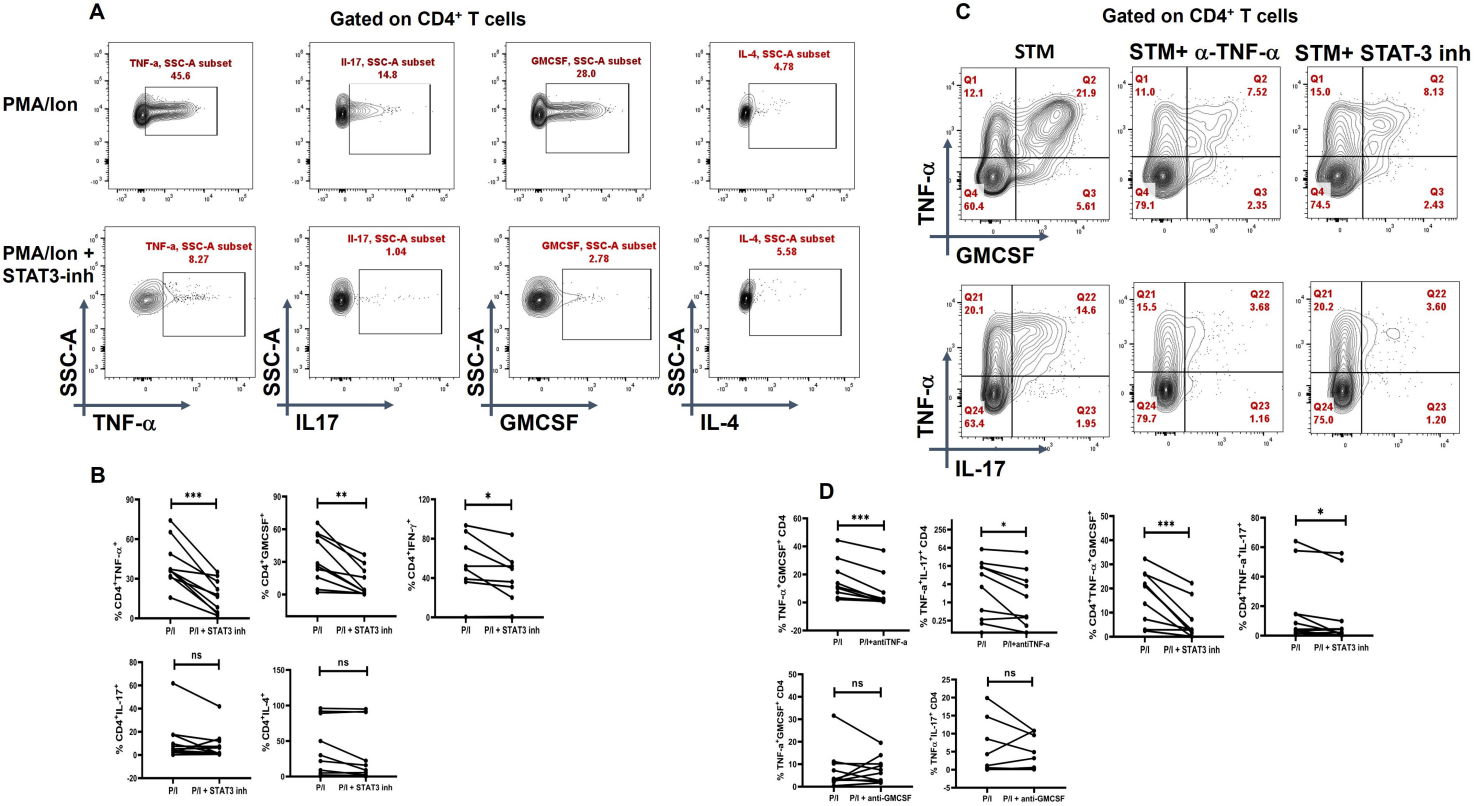
Modulation of cytokines with STAT-3 inhibitor treatment. Representative figures show significant decrease in pro-inflammatory cytokine expression with STAT-3 inhibitor treatment in CD4^+^ T cells (*n*=10) derived from PBMCs of T2DM patients **(A, B)**. In addition, CD4^+^ T cells also displayed a significant decrease in dual cytokine positivity (TNF-^+^GMCSF^+^ and TNF-^+^IL-17^+^) in both anti-TNF-α and STAT-3 inhibitor treatment **(C, D)**. The error bar in the above bar diagrams indicates SD. Paired t-Test was performed to compare the two groups **(B, D)**; *p* < 0.05 was considered statistically significant (*); *p* < 0.01 was considered to be very significant (**); *p* < 0.001 was considered to be highly significant (***). ns, not significant.

In our next objective, we wanted to examine whether TNF-α has any direct correlation with STAT-3 in eliciting the pro-inflammatory response. Previous studies have suggested that TNF-α expression may be associated with altered pSTAT3 levels in other chronic autoimmune conditions, such as rheumatoid arthritis (38). Thus, we evaluated the effect of pSTAT3 inhibition on cytokine expression wherein treatment with a pSTAT3 inhibitor resulted in a significant decrease in TNF-α along with other pro-inflammatory cytokines such as IFN-γ and GM-CSF (**Figures 7A, B**). We also report a decrease in cytokine secretion from the aberrant TNF-α^+^GM-CSF^+^ and TNF-α^+^IL-17^+^ double-positive populations (**Figures 7C, D**), with no significant changes observed in IL-17 alone population (**Figure 7B**).

### TNF-α modulates T-helper cell differentiation

**Figure 8:**
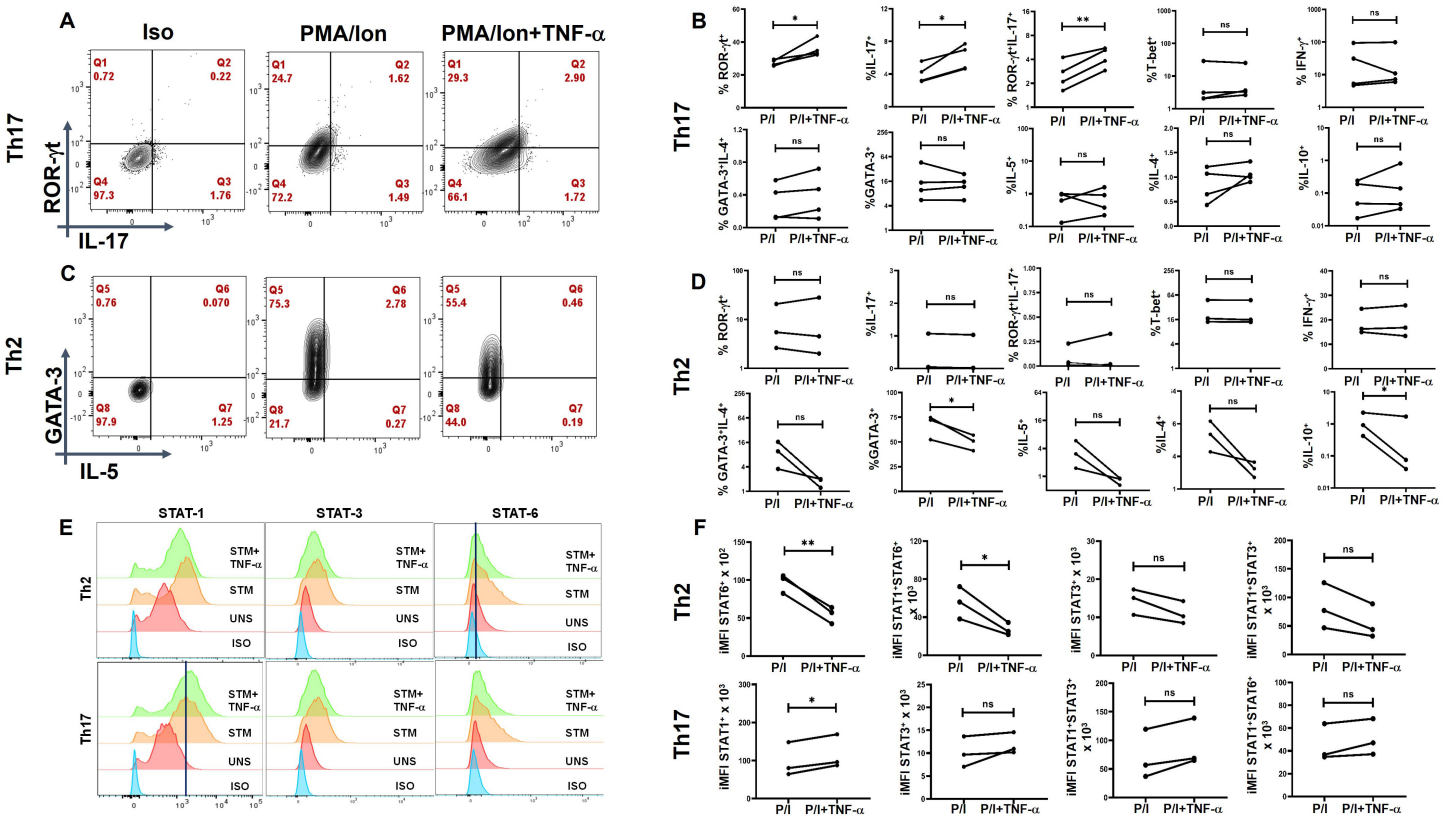
TNF-α modulates signature cytokine, transcription factor and pSTAT expression in *ex-vivo* differentiated human Th2 and Th17 subsets. Negatively isolated CD4^+^ T cells derived from PBMCs were activated with αCD3/28 stimulation, polarizing cytokines and neutralizing antibodies for 7 days, characterized for Th17 (*n*=4) and Th2 (*n*=3) phenotype and examined for altered expression of cytokines and transcription factors in the presence of TNF-α with PMA/Ionomycin restimulation. Th17 cells exhibited significantly elevated levels of RORγt, IL-17 and the dual RORγt^+^IL-17^+^ expression **(A, B)** while Th2 displayed a marked decrease in GATA3 and IL-10 expression **(C, D)** as shown in the flow cytometric and graphical plots. Cumulative histogram overlay showing changes in % of pSTAT expression in Th2 (*n*=3) and Th17 cells (*n*=3) **(E)**. Graphical Plots displayed a significant increase in integrated median fluorescence intensity (iMFI) of pSTAT1 in Th17 and a decrease in iMFI of STAT6 and STAT1^+^STAT-6^+^ in Th2 in the presence of TNF-α during restimulation was observed **(F)**. Paired T Test was performed to compare between the two groups **(B, D, F)**, *p* < 0.05 was considered statistically significant (*), *p* < 0.01 was considered to be very significant (**). ns, not significant.

To validate the role of TNF-α modulates the inflammatory cytokine response and assess its potential clinical relevance, we conducted *ex-vivo* experiments using differentiated human T-helper cell subtypes, that is, Th1, Th2, and Th17 cells. Following differentiation, we reactivated these T-helper subsets in the presence of TNF-α and analyzed their cytokine production and associated transcription factors. Our results demonstrated a significant upregulation of IL-17 and ROR-γt expression in Th17 cells upon TNF-α addition (**Figures 8A, B**), with a marked increase in STAT-1 expression in response to TNF-α (**Figures 8E, F**), further supporting its role in driving Th17-mediated inflammation. We did not find any significant difference in Th1 cytokines or transcription factors with TNF-α addition (**Supplementary Figure S8**). However, TNF-α stimulation resulted in a significant downregulation of GATA-3 expression in Th2 cells (**Figures 8C, D**), which was accompanied by a corresponding decrease in STAT-6 levels (**Figures 8E, F**), suggesting a suppressive effect on the Th2 lineage. This finding indicates that TNF-α not only amplifies the pro-inflammatory Th17 response but also inhibits the Th2-associated anti-inflammatory pathway. Collectively, ex-*vivo* studies reinforce our hypothesis that TNF-α plays a pivotal role in skewing the immune response toward a heightened inflammatory state. By promoting Th17 differentiation while concurrently suppressing Th2-associated regulatory mechanisms, TNF-α may contribute to the dysregulated immune environment observed in conditions such as T2DM, potentially exacerbating inflammation-driven pathologies.

## Discussion

The pathogenesis of T2DM in the context of meta-inflammation and its cellular sources remains inadequately understood and is primarily defined more by clinically defined biochemical parameters (39). This study sought to integrate and analyze meta-inflammation along with the above parameters. We establish that CD4^+^ T cells are the primary cells driving meta-inflammation via TNF-α in T2DM patients through STAT3 and provide an inclusive characterization of chronic low-grade inflammation in T2DM patients.

The demographic and clinical profile in **Table 1** provides a comprehensive overview of the study population, highlighting key differences in metabolic and inflammatory markers between T2DM patients and healthy controls. The elevated HbA1c, blood glucose, and triglyceride levels in T2DM patients reflect typical metabolic dysregulation associated with diabetes. These parameters, combined with information on lipid profile, renal function, and medication use, lay the foundation for exploring the role of immune dysregulation and inflammatory processes in T2DM. In addition, BMI data (not shown) indicated a distribution across normal, mildly obese, moderately obese, and highly obese categories, with no consistent correlation between BMI and immune, biochemical, or antibody isotype parameters. Interestingly, some patients with high BMI (>25) exhibited normal glycation (HbA1c~5) values, while others with normal BMI (18-25) displayed a higher glycation index (HbA1c 7-10). Given this logical inconsistency, BMI was not assigned any direct biological or immunological significance in the present study.

Our Bio-Plex data demonstrated that approximately 60% of the analytes tested were significantly elevated in T2DM patients compared to healthy controls. Notably, multiple pro-inflammatory cytokines, including TNF-α, IL-17, and GM-CSF, exhibited statistically significant elevation, suggesting a complex network of cytokine-driven immune dysregulation mediating meta-inflammation in T2DM, as also shown in multiple reports (40, 41). Most importantly, our stratified cytokine and antibody isotype level analyses demonstrated that a clear basal inflammatory profile in T2DM patients was obvious despite Hb1Ac, PPG, lipid levels, and treatment regimens notwithstanding.

One of the most significant findings was the elevated levels of antibody isotype levels across T2DM patients indicative of sustained antigenic exposure of APC-T cell-B cell interaction. The analyses clearly implied that a positive or negative correlation between the above proteins and HbA1c can only be considered with much stricter conditions, including regimented HbA1c monitoring every 30 days with Immune, cytokine, antibody isotype, and so forth, monitoring. Considering that a secreted cytokine would probably last 3–5 days maximally in a human, HbA1c, the 90–120-day glucose dysregulation marker, would be more than a difficult divisor. As endocrinologists and diabetologists monitor carbohydrate, lipid, metabolism, and organ health preferentially, the risk of an inflamed immune system is often overlooked. Nevertheless, our previous publication (14) and the present data clearly demonstrate that the secreted cytokine or chemokine is taken up by immune cells to affect deleterious immune effects in T2DM patients.

Not surprisingly, higher levels of anti-inflammatory cytokines, viz. IL-4, IL-5, and IL-10 suggested a counter to meta-inflammation, not necessarily secreted by the inflamed T2DM T-helper cells. Thus, our studies demonstrate that while T cells were or may not be major producers of anti-inflammatory cytokines, they were refractory to them, indicating that only T2DM B cells could and were indeed responsive. While the exact antigenic drivers were not identified here, similar non-protective or autoreactive antibody patterns have been described in metabolic and autoimmune conditions (14, 42), supporting the idea that humoral responses may contribute to the inflammatory *milieu*. These findings also align with emerging evidence suggesting that meta-inflammation may arise from aberrant interactions between innate immune cells and T-helper cells (43, 44), with the latter potentially serving as key regulators in initiating and sustaining this inflammatory cascade (45, 46).

Among the pro-inflammatory cytokines, TNF-α emerged as a central player in the inflammatory response in T2DM patients, consistent with its established role in promoting insulin resistance and contributing to diabetic complications such as nephropathy, neuropathy, and retinopathy (47–49). Flow cytometry analysis indicated CD3^+^CD4^+^ T-helper cells were the primary source of pro-inflammatory cytokines in PBMCs from T2DM patients, including TNF-α. Notably, even in “resting” T cells, cytokines such as TNF-α, GM-CSF, and IL-17 were secreted without stimulation, suggesting an “activated” CD4^+^ T-cell population that perpetuates inflammation. TNF-α secretion from effector T cells appeared as a non-specific flare-up, while GM-CSF and IL-17 production by memory T cells suggested a non-specific recall response, potentially directed toward self-antigens. The above findings give credence to the mandatory annual clinical check-ups for diabetics that include extensive examination for vision, cardio, pulmonary, sensory, renal, and so forth, as any underlying inflammation can initiate, propagate, and/or exacerbate organ damage. Dysregulated glucose and-or HbA1c control have been correlated with progressive damage to previously inflamed organs (9, 50, 51), and we hypothesize that this underlying meta-inflammation is driven by naïve and/or activated T-helper cells.

This study also highlights a significant association between dysregulated biochemical parameters, particularly elevated HbA1c levels, and pro-inflammatory cytokine profiles in T2DM patients. Across three distinct groups categorized by HbA1c levels—DM1 (6-8), DM2 (8-10), and DM3 (>10)—a progressive increase in cytokine signatures, including IFN-γ, TNF-α, and IL-17, was observed. These findings suggest even low-level chronic hyperglycemia exacerbates systemic inflammation by upregulating pro-inflammatory cytokine production. It also reinforces the importance of stringent glycemic control to mitigate immune dysregulation and chronic low-grade inflammation, which are hallmarks of T2DM pathophysiology. These results align with previous studies suggesting that sustained T-cell activation and cytokine production are critical drivers of systemic inflammation and auto-inflammatory processes in metabolic disorders (52, 53).

Activated CD4^+^ T-cell population demonstrated an exaggerated cytokine response, marked by TNF-α, GM-CSF, IL-17, and IFN-γ, which most probably contributes to organ damage. Both effector and memory T cells showed increased cytokine responses, reflecting underlying flare-ups and recall reactions, accompanied by notably low levels of anti-inflammatory cytokines, particularly IL-10. The elevated cytokine levels in T-cell compartments were significantly higher than those in healthy controls, suggesting that dysregulated glucose metabolism may alter baseline T-cell cytokine profiles, potentially driving excessive inflammation during infections. While IL-4 and IL-5 responses varied and seemed to be refractory in T cells, we hypothesize a potential unproductive antibody production through B cells and a protracted role in wound healing. Most importantly, our studies suggest prolonged higher levels of these cytokines for B cells to differentiate into plasma B cells, as evinced from our previous study (14) and others as well (53). These findings require further investigation in a larger cohort to clarify their role in T2DM.

TNF-α, a pleiotropic cytokine secreted by multiple sources, exhibits pleiotropic effects across various cell types, and plays a key role in regulating cellular function and inflammation. TNF-α can signal through TNFRI, which is widely expressed, and TNFRII, which is mostly limited to immune cells. TNF-α signaling via TNFRI induces inflammation and tissue degradation by activating NF-κB and MAPK pathways, which subsequently activate STAT1 and STAT3 (54). TNFRII, in contrast, is associated with cellular survival and proliferation via STAT5 activation (55). Collectively, these findings suggest that TNF-α signaling through TNFRI is the major driver for eliciting a pro-inflammatory response in T2DM.

Mechanistic analyses in our study revealed elevated expression of phosphorylated STAT1 and STAT3 in T2DM patients, where these phosphoproteins were also implicated in the pathogenesis of autoimmune diseases such as rheumatoid arthritis, systemic lupus erythematosus, and psoriasis. Confocal microscopy confirmed significantly higher nuclear co-localization of phosphorylated STAT1 and STAT3, further linking these pathways to the pro-inflammatory cytokine response in T2DM. Cytokines such as IFN-γ, IL-6, IL-18, and IL-1β, along with TNF-α and IL-17, are reported to be increased in pathology with concomitant activation of STAT1 and STAT3, with a majority being activated by TNFRI. TNFRI levels, as shown by Bio-Plex analysis was also found to be elevated because of TNF-α, a complementary mechanism of the immune system to sequester increased levels of TNF-α. These findings support the critical role of STAT3 in TNFRI-mediated inflammation and tissue degradation.

Inhibiting TNF-α resulted in a promising reduction in pro-inflammatory cytokines, including GM-CSF, IL-17, and IFN-γ, and decreased the occurrence of TNF-α^+^GM-CSF^+^ and TNF-α^+^IL-17^+^ cells. Additionally, TNF-α inhibition enhanced anti-inflammatory cytokine responses, such as IL-4 secretion. Anti-GM-CSF antibody and pSTAT5 inhibitor did not significantly affect inflammation, suggesting the primary and pathological involvement of the pSTAT1 and pSTAT3 pathways. While STAT1 inhibition yielded mixed results without statistical significance (**Supplementary Figure S9**) in 10 T2DM patients, pSTAT3 inhibition produced similar reductions in pro-inflammatory cytokines as observed with TNF-α inhibition. However, it did not show any change with anti-inflammatory cytokine secretion, possibly due to STAT3’s role in IL-10 regulation. A large study cohort might be needed to better understand the role of phospho STAT’s in driving inflammation in these patients. Clearly, immune monitoring wherein medication use is directed to regulating immune responses, inflammatory markers, and so forth, is critically lacking.

Our findings demonstrate that TNF-α plays a critical role in modulating T-helper cell differentiation, favoring a pro-inflammatory immune environment. The observed upregulation of IL-17 and ROR-γt expression in Th17 cells, along with increased STAT-1 activation, suggests that TNF-α is a key driver of Th17-mediated inflammation. This is consistent with previous studies indicating that TNF-α enhances Th17 polarization, contributing to chronic inflammatory diseases such as T2DM, RA, and inflammatory bowel disease (43, 56, 57). Conversely, the downregulation of GATA-3 and STAT-6 in Th2 cells suggests that TNF-α suppresses Th2 differentiation, thereby limiting anti-inflammatory responses. This suppression of Th2-associated regulatory mechanisms aligns with reports indicating that TNF-α can inhibit IL-4-driven Th2 polarization (58). Given the reciprocal regulation between Th17 and Th2 pathways, our results reinforce the notion that TNF-α skews immune homeostasis toward a sustained pro-inflammatory state, which may exacerbate metabolic inflammation in T2DM. This imbalance in T-helper cell subsets likely contributes to the low-grade systemic inflammation characteristic of T2DM, where elevated TNF-α levels correlate with insulin resistance and metabolic dysfunction (59–61). Our data suggest that TNF-α-induced Th17 expansion may sustain chronic inflammation, leading to persistent immune activation and tissue damage. Furthermore, the suppression of Th2 differentiation may impair regulatory mechanisms that normally counterbalance inflammation, further exacerbating immune dysregulation. Given these findings, targeting TNF-α signaling could be a therapeutic strategy to restore immune balance in T2DM and other chronic inflammatory conditions. TNF-α inhibitors have been shown to reduce systemic inflammation in autoimmune diseases, and their potential role in modulating T-helper cell differentiation in metabolic disorders warrants further investigation (62, 63). Future studies should explore whether blocking TNF-α signaling can shift the Th17/Th2 balance, thereby mitigating inflammation-driven metabolic dysfunction. In conclusion, our study highlights TNF-α as a key regulator of T-helper cell differentiation, promoting Th17-driven inflammation while suppressing Th2-associated anti-inflammatory responses. This imbalance may contribute to chronic inflammation in T2DM, emphasizing the need for targeted interventions aimed at modulating TNF-α activity.

A few limitations of the study include disparity in the age gap between T2DM patients and healthy controls. The male-to-female ratio differs slightly between groups, with a higher percentage of males in the T2DM group. This difference could affect outcomes, as there are known sex-based variations in immune response and metabolic regulation. Most T2DM patients are managed with oral hypoglycemic agents, with a smaller percentage on insulin. However, our findings clearly indicate that glucose or lipid dysregulation control alone through medication was not the best indicator of good immune homeostasis. Clearly, immune monitoring wherein medication use is directed to regulating immune responses, inflammatory markers, and so forth, is critically lacking. Ongoing research in our laboratory aims to characterize the unique T-helper phenotypes further, signaling alterations in T2DM, reduction in inflammatory markers with treatment, and so forth, over a year with a 90–120-day interval.

Our study provides a comprehensive characterization of chronic low-grade inflammation in T2DM patients. We identified that CD4^+^ T cells are primary cells driving meta-inflammation and observed higher levels of multiple pro-inflammatory cytokines, including TNF-α, IL-17, and GM-CSF in both “resting” and “activated” T cells, however in different T cell compartments. We observed a progressive increase in cytokine signatures across elevated HbA1c groups in T2DM patients. Further mechanistic analyses showed elevated expression of phosphorylated STAT1 and STAT3 in T2DM patients. Upon inhibiting TNF-α and STAT3, the levels of pro-inflammatory cytokines decreased, revealing their modulatory roles in regulating inflammation in T2DM CD4^+^ cells. This study clearly calls for immune profiling of T2DM patients along with the known and established health parameters for better diabetes management. Ongoing research in our laboratory aims to characterize the unique T-helper phenotypes further, signaling alterations in T2DM, reduction in inflammatory markers with treatment, and so forth, over a year with a 90–120-day interval. Further research could explore the therapeutic potential of pSTAT inhibitors in reducing meta-inflammation in T2DM.

## Data Availability

The original contributions presented in the study are included in the article/**Supplementary Material**. Further inquiries can be directed to the corresponding authors.
