# Supplementary material for: Meta-inflammation in type 2 diabetes mellitus: unveiling the role of aberrant CD4+ T cells and pro-inflammatory cytokine networks
Source: Front Immunol. 2025 Sep 15;16:1603484. doi: 10.3389/fimmu.2025.1603484 (PMC12477003; doi:10.3389/fimmu.2025.1603484)
Supplement: Supplementary file 1 [file DataSheet1.pdf]

Supplementary Figure 1

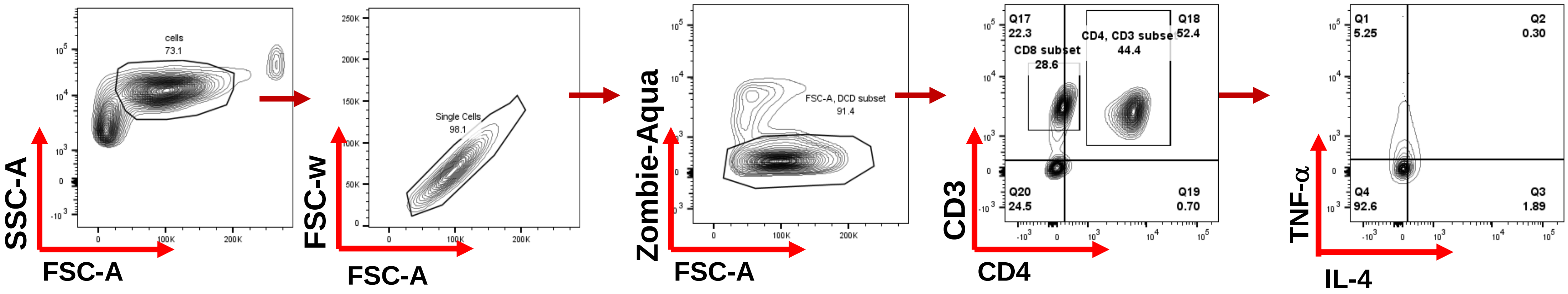

Supplementary Figure 1- Gating strategy for DM and HC PBMC derived CD4$^+$ T cells. Representative flow cytometry plots depict the gating strategy for all T2DM and HC CD4$^+$ T cell based experiments. Lymphocyte population is gated first, and then the singlet population is gated. Subsequently, live cells are gated based on staining of dead cells using Zombie Aqua dye (Biolegend). The dual positive CD3$^+$CD4$^+$ T cell population is eventually selected and gated on the total live cells and considered for further analysis of surface or intracellular protein markers.

Supplementary Figure 2

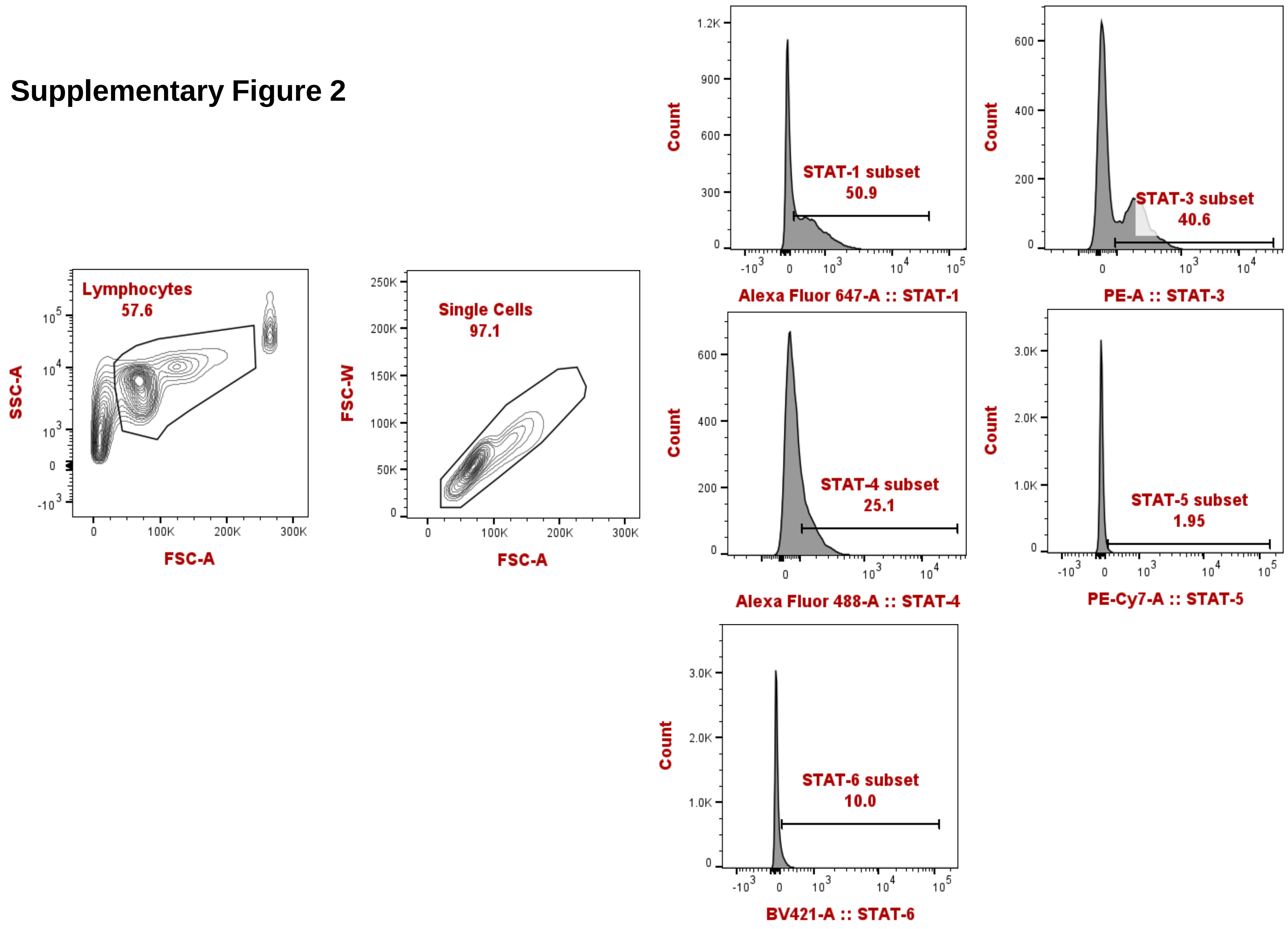

Supplementary Figure 2- Gating strategy for pSTAT expression in isolated CD4⁺ T-cells. Representative flow cytometry plots show gating strategy used to analyse phospho STATs from both DM and HCs. Lymphocyte population is gated first, and then the singlet population is gated. Subsequently, these cells were gate for different pSTAT proteins including pSTAT1, pSTAT3, pSTAT4, pSTAT5, pSTAT6.

Supplementary Table- 1: Reagents and Software used in the study

Reagent	Catalogue No.
Dynabeads™Untouched Human CD4 T cells Kit	Invitrogen<br>11346D
BD Cytofix/Cytoperm™	BD Biosciences<br>554714
eBiosciences™FoxP3/Transcription Factor Staining Buffer Set	Invitrogen<br>00-5523-00
Poly-L-Lysine0.01	SigmaAldrich<br>P4707
ProLong™Gold AntifadeMountantwith DAPI	Invitrogen<br>P36935
RPMI 1640	PAN-BIOTECH<br>P04-16520
FetalBovine Serum	PAN-BIOTECH<br>P30-1402
Human TruStain FcX™ (Fc Receptor Blocking Solution)	Biolegend-<br>422302
Zombie VioletTMFixable Viability Kit	Biolegend<br>423113
Human ProcartaPlex Mix & Match 46-Plex kit	Invitrogen<br>46-MX324DE
ProcartaPlex Human Antibody Isotyping Panels	Invitrogen<br>EPX070-10818-901
Purified Goat anti-Mouse IgG	Biolegend<br>405301
Phorbol-12-myristate (PMA)	Sigma<br>P8139-1MG
Ionomycin	Sigma<br>I0634-1MG
Brefeldin (BFA)	Sigma<br>B6542-5MG
Monensin	Biolegend<br>420701
Formaldehyde	Sigma<br>F8775-500 mL
Methanol	Sigma<br>646377-1L
Triton-X	Sigma<br>T8787-250 mL
DPBS, w/0: Ca and Mg	PAN BIOTECH<br>P04-36500
FlowJOVersion10.8	BD Biosciences
GraphPad Prism9	Dotmatics Pvt ltd.

Supplementary Table- 2: Neutralizing antibodies and Inhibitors used in the study

**Reagents**	**Catalogue No.**
In-Vivo SIM anti-Human TNF-α	BIOXCEL<br>SIM0006 (100ug)
Ultra-LEAF™ Purified anti-human GM-CSF Antibody	Biolegend<br>502206 (100ug)
Stat3 Inhibitor VIII, 5,15-DPP	Santa Cruz<br>Sc-204305 (Conc used- 5uM)
Fludarabine (STAT-1 inhibitor)	R&D systems<br>3495 (Conc used- 2.5uM)

Supplementary Table- 3: Fluorochrome-tagged antibodies used for flowcytometry

**Antibody**	**Catalogue No.**	**Isotype**
STAT6 BV421	Biolegend<br>686020	Biolegend<br>400429
STAT1 AF647	Biolegend<br>686412	BD Biosciences<br>557732
STAT5 PE-Cy7	Invitrogen<br>25-9010-42	Invitrogen<br>25-4714-80
STAT3 PE	Biolegend<br>698906	Biolegend<br>400322
STAT4 AF488	BD Biosciences<br>558136	BD Biosciences<br>558716
T-bet BV786	BD Biosciences<br>564141	BD Biosciences<br>563330
FOXP3 PE-Cy7	Invitrogen<br>25-4776-42	Invitrogen<br>25-4321-82
Ror-γT BV650	BD Biosciences<br>563424	BD Biosciences<br>563437
Ror-γt PE	Invitrogen<br>12-6988-80	BDBiosciences<br>557714
GATA-3 BV421	Biolegend<br>653813	Biolegend<br>400341
T-bet PE-Cy7	Invitrogen<br>25-5825-82	Invitrogen<br>25-4714-80
IL-4 BV605	Biolegend<br>500827	Biolegend<br>400433
IL17 BV711	Biolegend<br>512327	BD Biosciences<br>563044
IL-17 PE	BD Biosciences<br>560486	BD Biosciences<br>551436
IL-17 PerCPCy5.5	BD Biosciences<br>560799	BD Biosciences<br>552834

CD4 PECy7	Biolegend<br>300512	Invitrogen<br>25-4714-42
CD4 FITC	BD Biosciences<br>566320	Invitrogen<br>11-4714-41
CD4 PerCP-Cy5.5	Biolegend<br>300530	BD Biosciences<br>552834
IL-10 PE	BD Biosciences<br>559330	BD Biosciences<br>559317
IL-10 PerCP-Cy5.5	Biolegend<br>501418	Invitrogen<br>45-4301-80
Il-10 PE-Cy7	Biolegend<br>501419	BD Biosciences<br>557645
IFN-γ AF647	BioLegend<br>502516	BD Biosciences<br>557783
IL-5 BV421	Biolegend<br>504311	BD Biosciences<br>562868
IL-13 APC	Biolegend<br>501908	BD Biosciences<br>554686
CD45RO PECF594	BD Biosciences<br>562299	BD Biosciences<br>563484
CD45RO BV605	BD Biosciences<br>562790	BD Biosciences<br>562778
TNF-α BUV395	BD Biosciences<br>363996	BD Biosciences<br>563547
CD3 AF700	Biolegend<br>317340	Biolegend<br>400248
GMCSF PECF594	BD Biosciences<br>562857	BD Biosciences<br>562302
CD45RA BUV496	BD Biosciences<br>741182	BD Biosciences<br>612958

Supplementary Figure 3

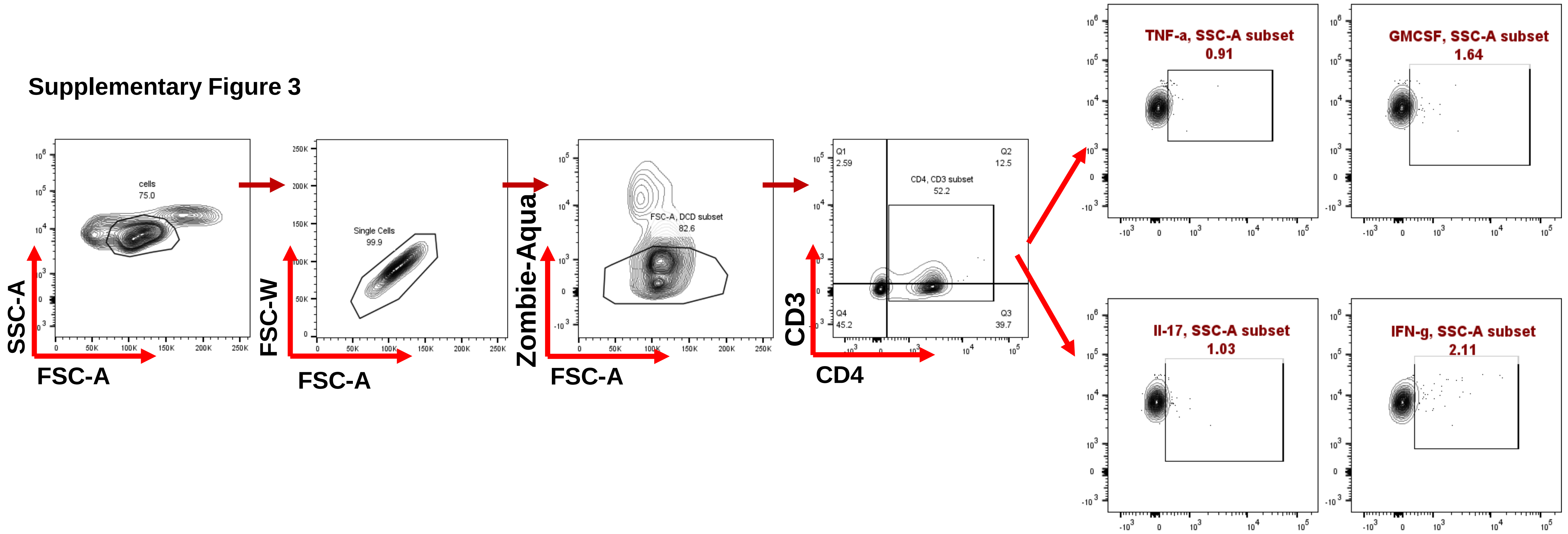

Supplementary Figure 3- Gating strategy for inhibitor studies. Representative flowcytometry plots show gating strategy used for all inhibitor experiments. Lymphocyte population is gated first, and then the singlet population is gated. Subsequently, live cells are gated based on staining of dead cells using Zombie Aqua dye (Biolegend). The dual positive $CD3^{+}CD4^{+}$ T cell population is eventually selected and gated on the total live cells and considered for further analysis of surface or intracellular protein markers

Supplementary Figure 4

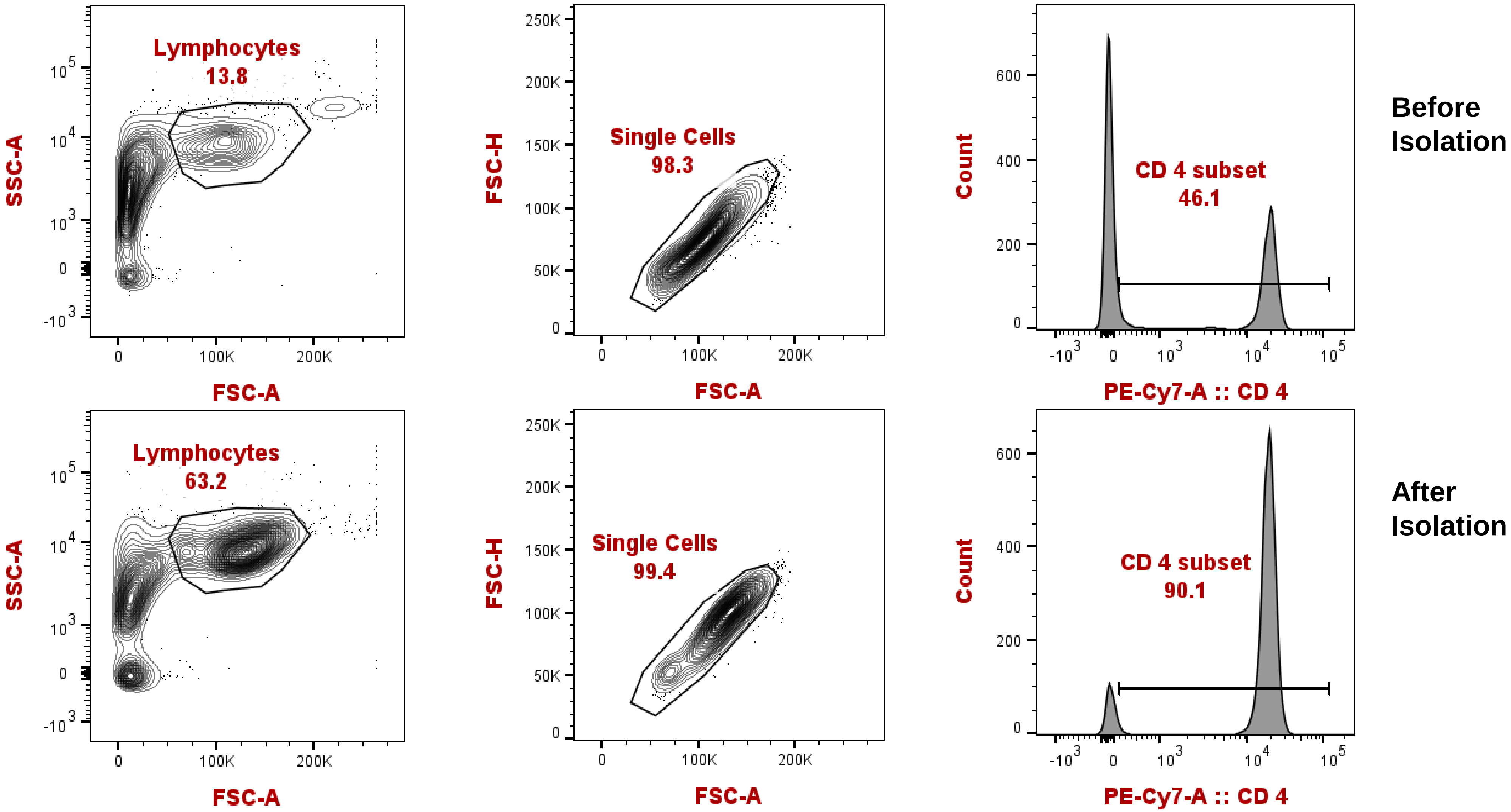

Supplementary Figure 4: $CD4^+$ T cell purity validated for *ex vivo* experiments. Representative flow cytometry plot shows the purity of $CD4^+$ T cells derived from PBMC of healthy controls confirmed prior to initiation of Th2 and Th17 polarization.

Supplementary Figure 5

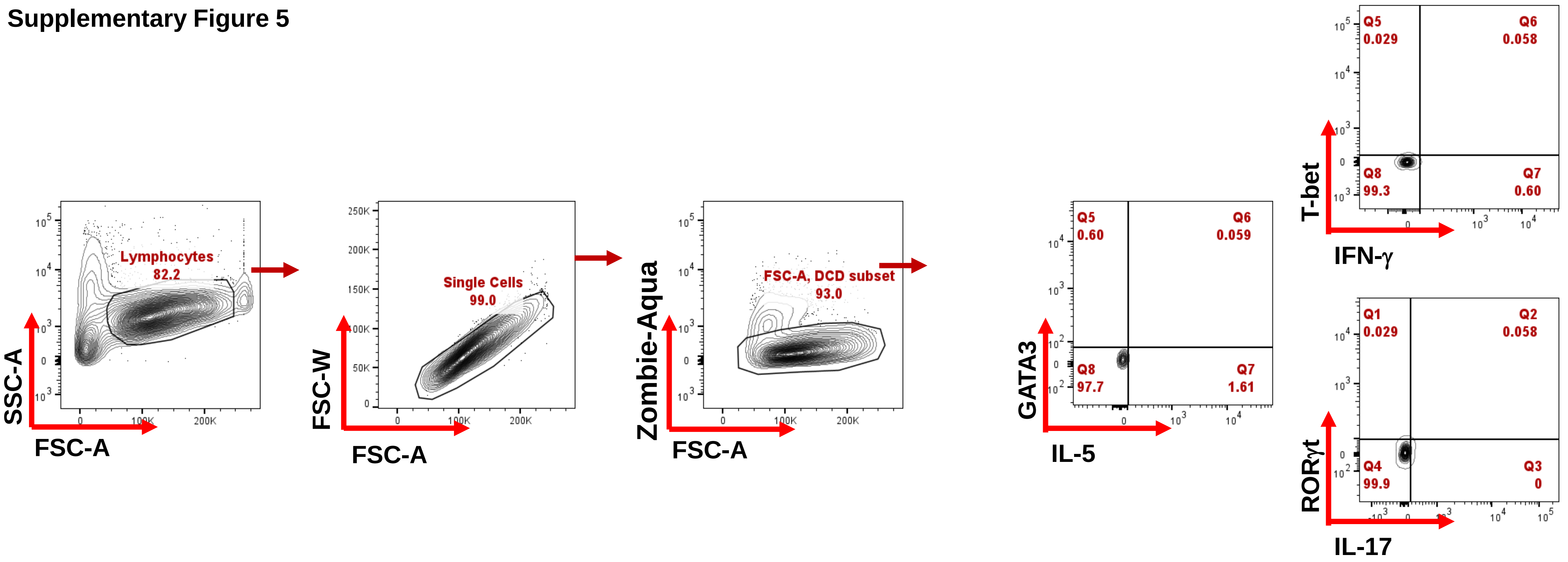

Supplementary Figure 5- Gating strategy for *ex-vivo* studies. Representative flowcytometry plots show gating strategy used for all *ex-vivo* experiments. Lymphocyte population is gated first, and then the singlet population is gated. Subsequently, live cells are gated based on staining of dead cells using Zombie Aqua dye (Biolegend). The dual positive population of transcription factors and cytokines is eventually selected and gated on the total live cells and considered for further analysis

Supplementary Figure 6

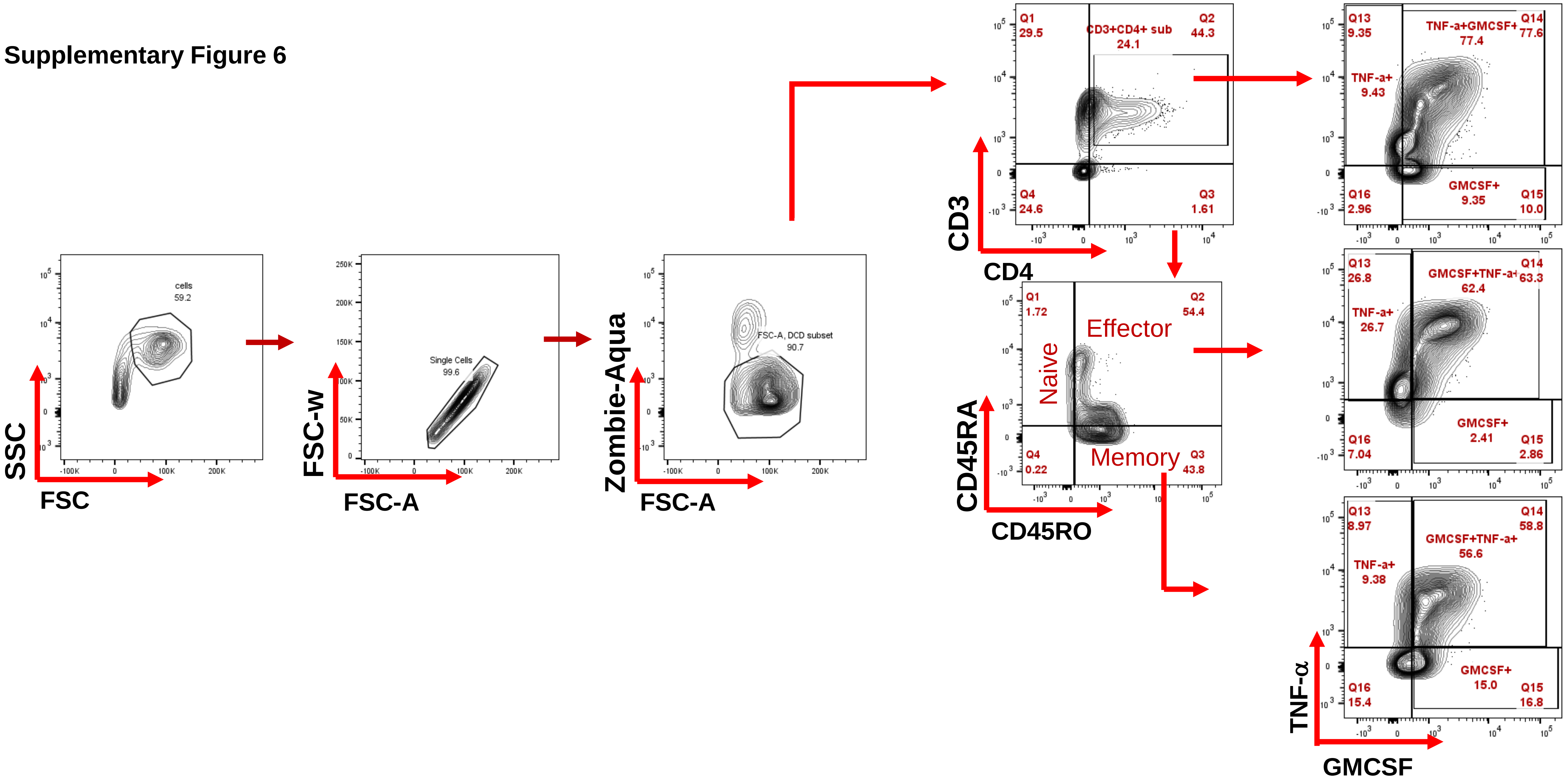

Supplementary Figure 6- Gating strategy to determine cytokines from CD4^{+}T-cell compartments from PBMCs of DM patients. Representative flow cytometry plots shows the gating strategy done for all T2DM patients to analyze the different cytokines secreted through the T-cell compartments. Breifly gating was done on lymphocytes followed by singlets. Subsequently, live cells are gated based on staining of dead cells using Zombie Aqua dye (Biolegend). Further gating was done on CD3^{+}CD4^{+} double positive cells to analyse on CD4 helper T-Cells. Next, T-cell compartment analysis was done on gating based on surface markers CD45RA and CD45RO. Cytokines were then analysed on Naïve (CD45RA^{+}RO^{-}), Effector (CD45RA^{+}RO^{+}), Memory (CD45RA^{-}RO^{+}).

Supplementary Figure 7 A

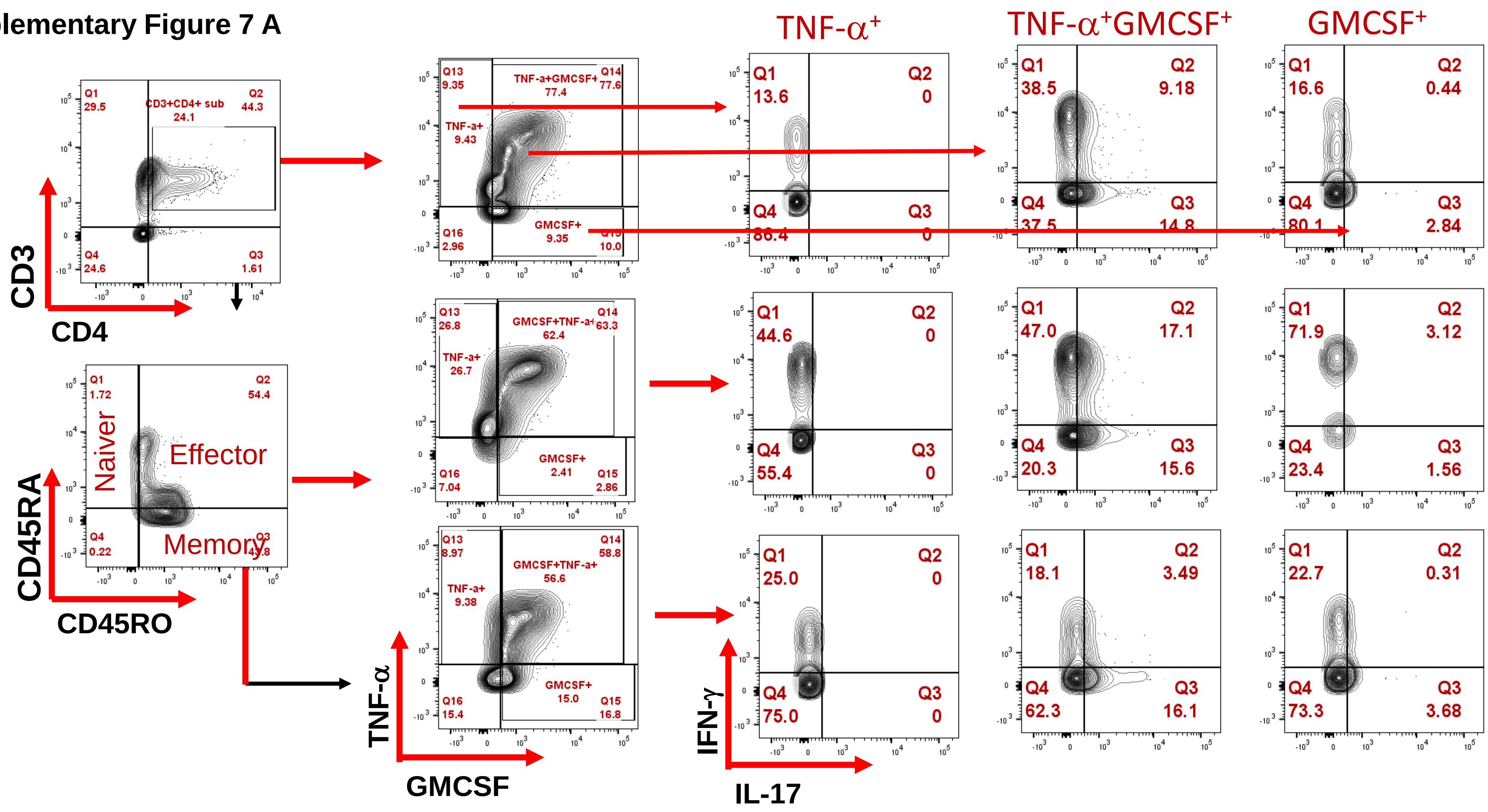

Supplementary Figure 7 A- Multiple cytokine positive cells in DM. Representative flow cytometry plots show cytokines positivity from different T-cell compartments for TNF-α and GMCSF positive cells. This population were further analysed for 3 different cell types namely TNF-α+ GMCSF- cells, TNF-α+ GMCSF+ cells and TNF-α- GMCSF+ cells. The flow cytometric plots shows an increase in aberrant phenotype of cytokines positive for four different pro-inflammatory cytokines

Supplementary Figure 7 B

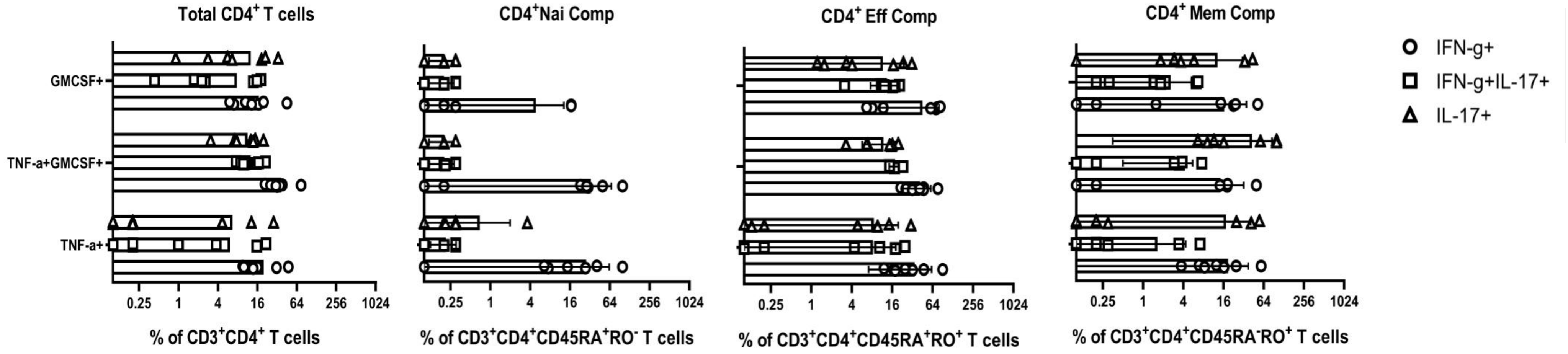

Supplementary Figure 7 B- Multiple cytokine positive cells in DM. Representative bar graph shows statistical comparision (Kruskal Walis test) of 3 different population of TNF-α and GMCSF positive for IFN-γ and IL-17 respectively.

Supplementary Figure 8

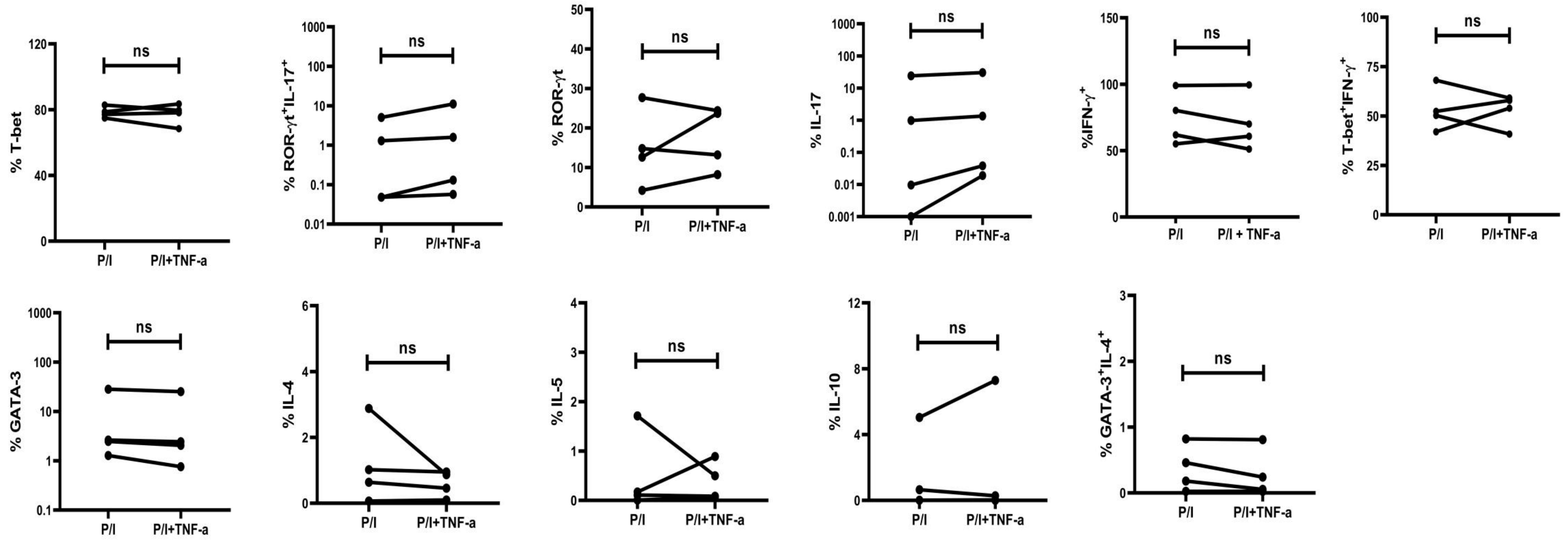

Supplementary Figure 8- Effect of TNF-α on terminally differentiated Th1 cells. Negatively isolated $CD4^+$ T cells derived from PBMCs were activated with αCD3/28 stimulation, polarizing cytokines and neutralizing antibodies for 5 days followed by resting for 2 days, characterized for Th1 (n=4) phenotype and examined for altered expression of cytokines and transcription factors in the presence of TNF-α with PMA/Ionomycin restimulation. Th1 cells did not display any change in levels of any cytokine or transcription factors. Representative bar graph shows statistical comparision (paired t Test) of 4 individual experiments

Supplementary Figure 9

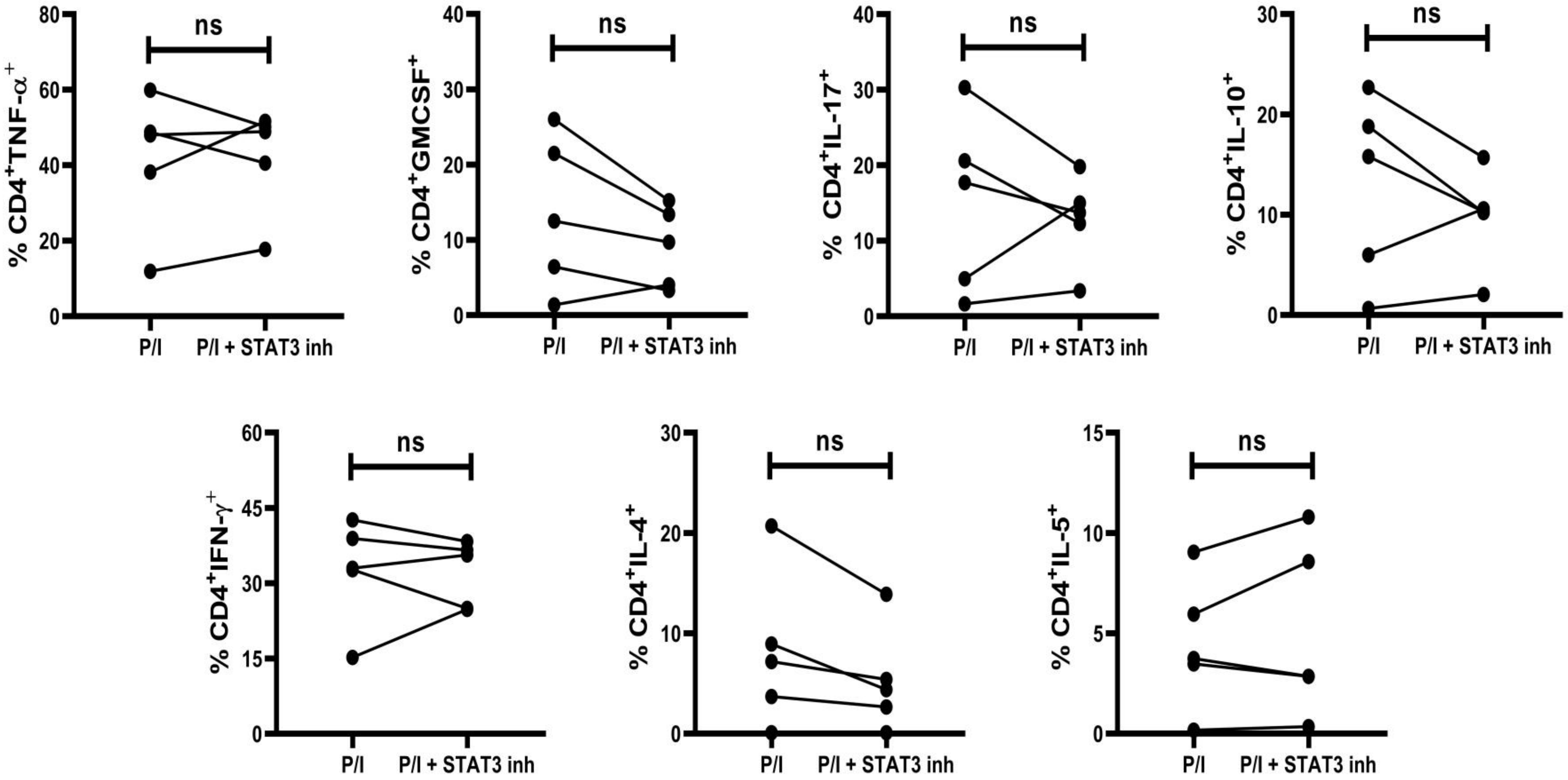

Supplementary Figure 9- Cytokine expression upon STAT1 inhibitor treatment. Representative figures showing modulation of pro-inflammatory and anti-inflammatory cytokine expression in T2DM PBMCs (n=5) gated on $CD4^+$ T cells with STAT1 inhibitor treatment along with PMA/Ionomycin stimulation. $CD4^+$ T cells did not show any significant difference of any cytokines as shown in the graphical plots. The error bar indicates SD. Paired t-Test was performed to compare the two groups; $p < 0.05$ was considered statistically significant (*); $p < 0.01$ was considered to be very significant (**); $p < 0.001$ was considered to be highly significant (***); $p < 0.0001$ was considered extremely significant (****). ns, not significant.
